# Structural studies of the yeast DNA damage-inducible protein Ddi1 reveal domain architecture of this eukaryotic protein family

**DOI:** 10.1038/srep33671

**Published:** 2016-09-20

**Authors:** Jean-François Trempe, Klára Grantz Šašková, Monika Sivá, Colin D. H. Ratcliffe, Václav Veverka, Annabelle Hoegl, Marie Ménade, Xin Feng, Solomon Shenker, Michal Svoboda, Milan Kožíšek, Jan Konvalinka, Kalle Gehring

**Affiliations:** 1 Groupe de Recherche Axé sur la Structure des Protéines, Department of Biochemistry, McGill University, 3649 Promenade Sir William Osler, Montreal, QC, H3G 0B1, Canada; 2 Gilead Sciences and IOCB Research Center, Institute of Organic Chemistry and Biochemistry of the Academy of Sciences of the Czech Republic, Flemingovo n. 2, 166 10 Prague 6, Czech Republic; 3 Department of Biochemistry, Faculty of Science, Charles University, Hlavova 8, 120 00 Prague 2, Czech Republic; 4 First Faculty of Medicine, Charles University in Prague, Katerinska 32, 121 08, Prague 2, Czech Republic; 5 Department of Physical and Macromolecular Chemistry, Faculty of Science, Charles University, Hlavova 8, 120 00 Prague 2, Czech Republic

## Abstract

The eukaryotic Ddi1 family is defined by a conserved retroviral aspartyl protease-like (RVP) domain found in association with a ubiquitin-like (UBL) domain. Ddi1 from *Saccharomyces cerevisiae* additionally contains a ubiquitin-associated (UBA) domain. The substrate specificity and role of the protease domain in the biological functions of the Ddi family remain unclear. Yeast Ddi1 has been implicated in the regulation of cell cycle progression, DNA-damage repair, and exocytosis. Here, we investigated the multi-domain structure of yeast Ddi1 using X-ray crystallography, nuclear magnetic resonance, and small-angle X-ray scattering. The crystal structure of the RVP domain sheds light on a putative substrate recognition site involving a conserved loop. Isothermal titration calorimetry confirms that both UBL and UBA domains bind ubiquitin, and that Ddi1 binds K48-linked diubiquitin with enhanced affinity. The solution NMR structure of a helical domain that precedes the protease displays tertiary structure similarity to DNA-binding domains from transcription regulators. Our structural studies suggest that the helical domain could serve as a landing platform for substrates in conjunction with attached ubiquitin chains binding to the UBL and UBA domains.

The ubiquitin system is primarily a signaling pathway whereby substrates tagged with various types of ubiquitin chains or ubiquitin-like (UBL) modifiers undergo different fates in the cell^1^. Ubiquitinated substrates are recognized by receptor proteins that contain ubiquitin-binding domains such as ubiquitin-interacting motifs (UIM) and ubiquitin-associated (UBA) domains^2^. In *Saccharomyces cerevisiae*, three ubiquitin receptors (Ddi1, Rad23, and Dsk2) have C-terminal UBA domains that bind ubiquitin and Lys48-linked polyubiquitin^3^,^4^,^5^,^6^. These proteins also bear an N-terminal UBL domain that binds Rpn1 in the 19S proteasome subunit^7^,^8^,^9^,^10^. Ddi1 and Rad23 are DNA-damage inducible proteins, and both have been shown to suppress the temperature sensitivity of a *pds1* mutant^11^. The protein Pds1 (securin) is a mitotic checkpoint control protein, and its ubiquitination by the anaphase-promoting complex (APC) and subsequent degradation is required for the separation of sister chromatids. The triple-deletion mutant Δ*ddi1*Δ*rad23*Δ*dsk2* shows a synthetic effect and delays in the onset of G2/M phase and anaphase, suggesting redundant roles in cell cycle progression^12^.

Over the last ten years, the biology of yeast Ddi1 has been investigated from different perspectives. The expression of the *DDI1* gene is controlled by a bidirectional DNA-damage inducible promoter that divergently transcribes *DDI1* and *MAG1*, a 3-methyladenine DNA glycosylase involved in a base-excision-repair pathway^13^,^14^. These two genes are differentially regulated in response to different DNA-damage checkpoint pathways^15^,^16^,^17^. Strong expression of *MAG1* and *DDI1* can thus be induced by the addition of methyl methane-sulfonate to yeast cells, which triggers the *CHK1*- and *MEC1*-dependent DNA-damage response (DDR) pathways. Recent studies also indicate a possible role for Ddi1 in degradation of the Ho endonuclease, the enzyme responsible for switching alleles at the mating type locus *MAT*^18^. Activation of the *MEC1*-dependent DDR pathway leads to the phosphorylation and rapid degradation of the Ho protein by the ubiquitin-proteasome system^19^. Phosphorylated nuclear Ho is exported to the cytoplasm *via* the Msn5 nuclear exportin and ubiquitinated by the SCF^Ufo1^ E3 ligase complex^20^. Interestingly, Ho accumulates in Δ*ddi1* cells, but not in Δ*rad23* or Δ*dsk2* cells^18^. This specificity was attributed to specific interactions between the UBL domain of Ddi1 and four tandem UIMs located at the C-terminus of Ufo1^21^. Ufo1 binds phosphorylated Ho through its F-box domain to mediate SCF-dependent ubiquitination^18^.

Ddi1 (also known as Vsm1 from v-SNARE-master 1) was independently identified as a SNARE-interacting protein in a yeast two-hybrid screen using the endocytic Snc2 protein as a bait^22^. Ddi1 interacts with both exo- and endocytic v-SNARE proteins (Snc1 and Snc2). Overexpression of Ddi1 in yeast bearing a mutation in the *sec9* gene (t-SNARE) inhibits protein secretion, suggesting that Ddi1 is a negative regulator of exocytosis. It was later shown that Ddi1 binds to the exocytic t-SNARE Sso1, which precludes binding of Sso1 to its functional partner Sec9 and thus inhibits exocytosis^23^. Binding of Ddi1 to Sso1 is promoted by phosphorylation of the N-terminal autoinhibitory domain of Sso1. The interaction is mediated by a linker region of Ddi1 located between the protease and UBA domains. The linker includes a PEST motif and a phosphorylation site (T348) that also regulates exocytosis^24^. Consistent with these findings, Δ*ddi1* yeast cells show increased global protein secretion^23^,^25^. Ddi1 is also required for endocytosis of the guanine nucleotide-binding protein Gα^26^. Overall, these various studies point towards a role for Ddi1 in cell cycle and growth control, as well as protein trafficking.

Yeast Ddi1 has three structural domains. It has an N-terminal UBL domain that shares only 14% sequence identity with ubiquitin. Its central retroviral protease-like domain (RVP), which is common to all eukaryotic Ddi1 orthologs, is homologous to retroviral aspartic proteases^27^. The active site aspartate is required for repression of protein secretion in yeast^25^, and this phenotype can be inhibited by HIV protease inhibitors^28^, strongly suggesting that this function of Ddi1 is linked to its protease activity. The Ddi1-like protein from *Leishmania major* displays proteolytic activity at acidic pH^29^, suggesting that the protein may be active only in acidic vesicular compartments. The three-dimensional structure of the isolated protease domain confirms that the domain adopts the typical aspartyl protease fold, with a two-fold dyad symmetry that allows Asp220 from two subunits to form hydrogen bonds with a catalytic water molecule^30^. Yeast Ddi1 also bears a C-terminal UBA domain that is found only in plants, fungi, and invertebrates, but not in vertebrate Ddi1 orthologs^3^,^4^,^5^. The UBA domain from the *Schizosaccharomyces pombe* ortholog Mud1 binds selectively to K48-linked diubiquitin (Ub_2_) through two ubiquitin-binding sites^5^. In spite of all these studies, the overall function and substrate(s) of Ddi1 protease domain remain elusive.

Here, we report findings from structural and functional studies of full-length Ddi1 from *S. cerevisiae*. We determined a new crystal structure of the RVP domain of Ddi1 that provides insight into its putative substrate recognition mechanism. We determined the solution structure of the UBL domain by NMR spectroscopy and performed interaction studies with different proposed ligands. We found that UBA and UBL both bind ubiquitin and that Ddi1 binds K48-linked diubiquitin with enhanced affinity. We also determined the structure of a new α-helical domain (named HDD) that precedes the RVP domain and could play a role in substrate recognition. We used SAXS to investigate the structure and dynamics of the module formed by the UBL, HDD and RVP domains. Finally, we performed Proteomic Identification of protease Cleavage Sites (PICS) analysis with full-length Ddi1 at both acidic and neutral pH to explore substrate specificity^31^.

## Results

### Crystal structure of the Ddi1 retroviral protease-like domain reveals a potential substrate-binding loop

**Figure 1:**
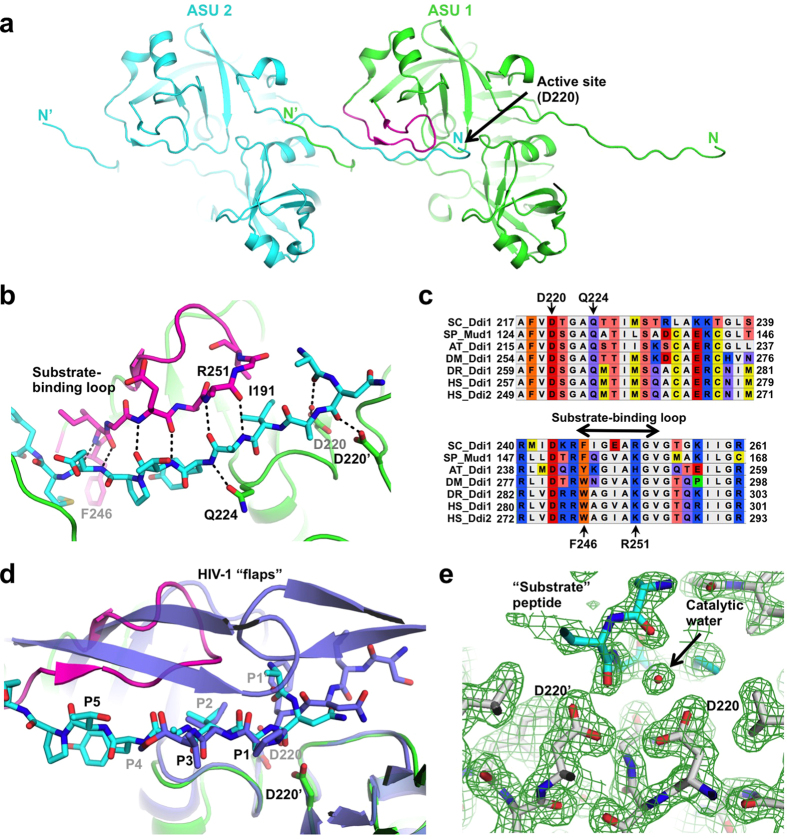
Crystal structure of the yeast Ddi1 protease domain reveals a potential substrate-binding mode. (**a**) Cartoon representation of two adjacent asymmetric units (ASU), showing the N-terminus of one molecule in ASU #2 (cyan) binding to the active site of a dimeric protease in ASU #1 (green). The loop that forms interactions with the N-terminal peptide is colored magenta. (**b**) Close-up view of the interaction between the N-terminal peptide and the active site. Hydrogen bonds are shown as dashed lines, and important residues are labeled. **(c)** Sequence alignment of Ddi1 orthologs from different species. SC, *Saccharomyces cerevisiae*; SP, *Schizosaccharomyces pombe*; AT, *Arabidopsis thaliana*; DM, *Drosophila melanogaster*; DR, *Danio rerio*; HS, *Homo sapiens*. (**d**) Superposition of the Ddi1 protease structure (green) with HIV-1 protease bound to a peptide substrate mimetic (violet, PDB 7HVP). The HIV substrate mimetic is shown in blue, and the Ddi1 pseudo-substrate N-terminal peptide is shown in cyan. (**e**) *2F*_*o*_*-F*_*c*_ electron density maps of the active site, revealing the position of a water molecule that could act as a potential nucleophile in a proteolytic reaction.

The most conserved segment of the Ddi1 family is its RVP domain, which probably defines its biochemical function. While the structure of the RVP had previously been determined^30^, the substrate-binding mode remained unknown. We obtained a new crystal structure of the RVP domain of Ddi1 (residues 185–325) at 1.8 Å resolution. The unit cell dimensions differ from those of the previously published structure. The structure was solved by molecular replacement and refined to *R*_*work*_*/R*_*free*_ of 18.3/21.3% (Supplementary Table 1). The asymmetric unit consists of two chains, which form a dimer with non-crystallographic C2 symmetry. The structure is very similar overall to the previous one, with a backbone rmsd of 0.6 Å for residues that are common to both structures. In the new structure, electron density is visible for the N-terminal segment spanning residues 185–199. Intriguingly, this N-terminal segment binds into the active site (Asp220) of an adjacent protease dimer in a different asymmetric unit (Fig. 1a). The segment is positioned such that the active site Asp220 might cleave between amino acids 189 and 190 (Fig. 1b). However, the protein does not undergo auto-proteolysis in solution; mass spectrometry confirmed that the RVP domain remains intact even under acidic conditions (Supplementary Fig. S1). Nonetheless, while the observed arrangement is a likely an artefact of crystallization, the N-terminal segment acts as pseudo-substrate and reveals how Ddi1 might engage a substrate. The N-terminus adopts a β-strand conformation that interacts extensively with a loop formed by residues 245–258 (Fig. 1b). This loop is only visible in the chain that docks the N-terminus. There are multiple hydrogen bonds between the backbone atoms of the pseudo-substrate and the loop, suggesting that it might be involved in positioning the substrate for catalysis. The side chain of the conserved Gln224 (Fig. 1c) also makes a hydrogen bond with the backbone amide of the pseudo-substrate (Fig. 1b). The side chain of Phe246, which is conserved as an aromatic residue across Ddi1 orthologs, interacts with a methionine in the N-terminal fragment (Fig. 1c), suggesting this may be a specificity determinant for the protease activity of Ddi1. Arg251 is also conserved and could potentially interact with a substrate, but we did not observe any electron density for its side chain, implying that it is disordered. Finally, the side chain of Ile191 fits snugly into a hydrophobic groove formed by the loop and the rest of the RVP domain.

Comparison with an HIV protease structure reveals substantial differences in substrate binding. In the structure of the HIV protease bound to a substrate-based hydroxyethylamine inhibitor^32^, two flaps wrap around the substrate analog (Fig. 1d). Main-chain amides in the flaps of HIV-1 protease form hydrogen bonds with a water molecule that also binds the substrate analog. In Ddi1, the flap does not wrap around the pseudo-substrate, thus leaving it solvent-exposed. Remarkably, the pseudo-substrate and analogs in both Ddi1 and HIV-1 adopt very similar conformations, with main-chain atoms in the same configuration for amino acids in the P1 to P3 positions. Notably, both Ddi1 and HIV-1 protease substrates have an isoleucine in the P2 position. Overall, this suggests that the catalytic mechanism employed by both enzymes is similar, although they may engage their substrates differently.

The structure also reveals how Ddi1 could cleave a peptide bond: the catalytic residue Asp220 holds an ordered water molecule in place, which may act as the nucleophile for peptide bond hydrolysis (Fig. 1e). The symmetry-related Asp220′ forms a hydrogen bond with a carbonyl in the P1′ position, rendering it more susceptible to nucleophilic attack. The water molecule is within hydrogen-bonding distance of two carbonyl oxygens in the N-terminal fragment, but it is not positioned in a way that would enable catalytic attack of the carbonyl carbon. Thus, the observed conformation would not lead to proteolysis. Thus, it remains unknown how the protease activity can be triggered and what the substrate(s) might be.

### Proteomics screen for substrate(s) of Ddi1 protease

To characterize putative substrate(s) of Ddi1 RVP, we used a proteomic technique that employs a proteome-derived peptide library as a proteolytic substrate screen^31^. We used a peptide library derived from haploid yeast cells. We analyzed full-length Ddi1 as well as its “inactive” D220A variant at pH 4.0, 5.0, and 7.4. The data analysis revealed no Ddi1-dependent cleavage at all pH tested, whereas the HIV-1 positive control produced significant amount of proteolysis (Supplementary Fig. S2). This suggests that the protease domain of Ddi1 requires activation or may cleave intact proteins in their native conformations.

### The UBL and UBA domains of Ddi1 bind to ubiquitin

**Figure 2:**
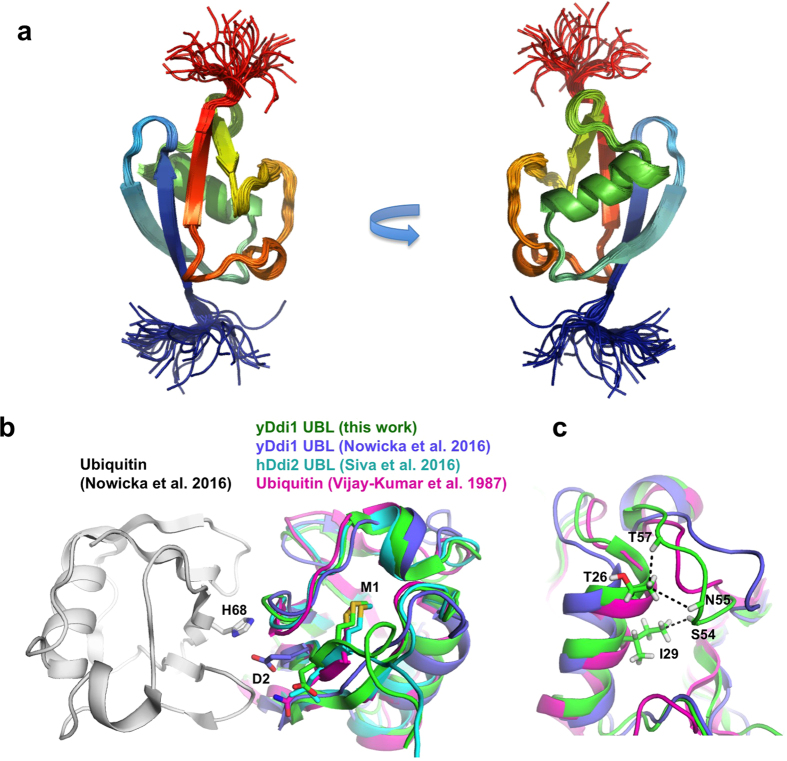
Solution structure of the Ddi1 UBL. (**a**) NMR solution structure of the UBL domain (a.a. 1–80; PDB 2N7E). An ensemble of 43 models is shown in cartoon representation, colored from blue to red from the N- to C-terminus. (**b**) Superposition of the yeast Ddi1 UBL NMR structure (green, pdb 2N7E) with the yeast Ddi1 UBL docked to ubiquitin (violet and white, pdb 2MWS), the human Ddi2 UBL NMR structure (cyan, pdb 2N7D), as well as the ubiquitin crystal structure (magenta, pdb 1UBQ). The side-chains of Met1 and Asp2 in the yDdi1 UBL (Gln2 and Leu2 in ubiquitin and hDdi2, respectively), and His68 in ubiquitin are shown as sticks. **(c)** Same as in (**b**), in a different orientation. The dashed lines indicate NOEs between Hα and methyl protons in the yeast Ddi1 UBL that confirms the proximity between the loop formed by a.a. 52–58 and the N-terminal segment of an a-helix (a.a. 26–29).

UBL domains are known to be protein:protein interaction modules, and thus could potentially play a role in protease substrate recognition. We thus characterized the structure and interactions mediated by the Ddi1 UBL domain using NMR. The domain adopts the ubiquitin fold (Fig. 2a,b and Supplementary Table 2) in spite of its low sequence similarity to ubiquitin. Yeast Ddi1 UBL has a rather shallow hydrophobic patch that is located at the same sequential and structural location as in human Rad23A^33^. This β-sheet patch potentially may be a protein-protein interaction site. However, NMR titrations of ^15^N-Ddi1 UBL with potential UIM-containing ligands Ufo1 and Rpn10 showed no chemical shift perturbations, as reported by others (Supplementary Fig. S3)^34^,^35^. Moreover, no significant chemical shift perturbations were observed with the addition of Ddi1 86–325 (helical domains + protease) or Ddi1 388–428 (UBA), suggesting that the UBL would make no intramolecular contacts with other Ddi1 domains in the context of the full-length protein.

During preparation of this manuscript, the structure of Ddi1 UBL was published and its interaction with ubiquitin reported^34^. Comparison of the structures revealed small but significant differences. In our structure, the loop spanning a.a. 52–58 is in proximity to the N-terminus of the α-helix formed by a.a 24–34, with unambiguous NOEs between the two segments (Fig. 2c). This conformation, similar to the one found in ubiquitin, is different in the yeast Ddi1 UBL structure previously reported, where it is more distant from the α-helix. Moreover, our construct includes the N-terminal Met1 residue, which was absent from the construct used by Nowicka *et al*. The main-chain carbonyl of Met1 makes a hydrogen bond with Val19 and extends the first β strand, and its side-chain is oriented towards the core of the domain, as in ubiquitin or in the human Ddi2 UBL domain^36^ (Fig. 2b). This confers a different orientation to Asp2, which side-chain points towards His68 in ubiquitin docked to yeast Ddi1 UBL^34^.

**Figure 3:**
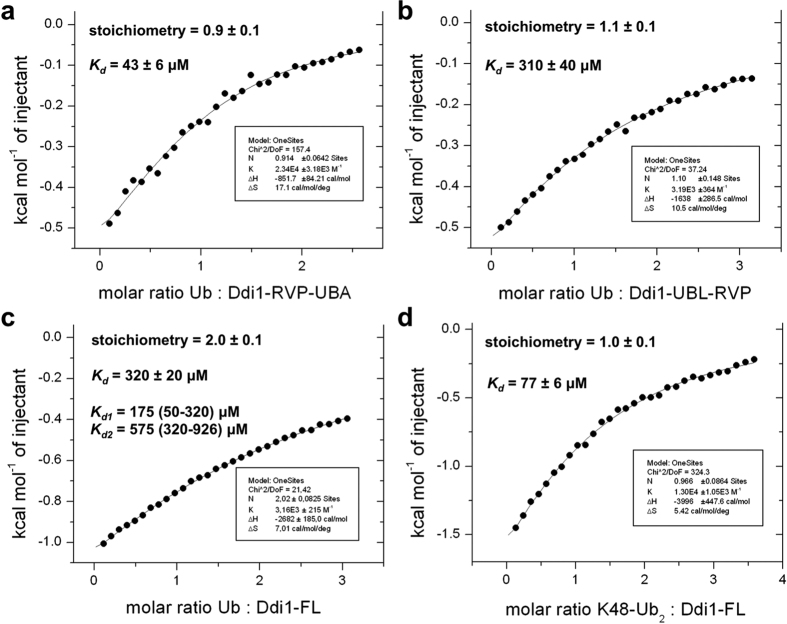
The UBL and UBA domains of Ddi1 bind to ubiquitin and diubiquitin. Ubiquitin or K48-linked diubiquitin (Ub_2_) were added by syringe to Ddi1 constructs in the sample cell. The different titrations were (**a**) ubiquitin to RVP-UBA, (**b**) ubiquitin to UBL-RVP, (**c**) ubiquitin to full-length Ddi1, and (**d**) K48-Ub_2_ to full-length Ddi1. For full-length Ddi1 binding to ubiquitin (**c**), the data was also fitted to a model with two independent binding sites (*K*_*d1*_ and *K*_*d2*_), each with a stoichiometry of 1.

To determine whether our Ddi1 constructs would bind to ubiquitin, we used isothermal titration calorimetry (ITC). We tested binding in the context of the full-length protein, with deletion of either the UBL or the UBA domain. Ubiquitin was found to bind the RVP-UBA construct (without the UBL) with a *K*_*d*_ of 43 μM, whereas it bound the UBL-RVP construct (without the UBA) with a *K*_*d*_ of 310 μM (Fig. 3a,b). Titration of full-length Ddi1 with ubiquitin yielded an average *K*_*d*_ of 320 μM, with a 2:1 stoichiometry (Fig. 3c). We also fitted the latter data to a model with two independent sites and found ranges of *K*_*d*_ values that are close to the values obtained for the deletion constructs. These results are thus consistent with both UBA and UBL interacting with ubiquitin. Finally, we found that K48-Ub_2_ binds full-length Ddi1 with a *K*_*d*_ of 77 μM and a 1:1 stoichiometry, suggesting that each Ddi1 dimer binds two K48-Ub_2_ molecules (Fig. 3d).

### The structure of the helical domain of Ddi1 reveals similarities to DNA-binding domains

**Figure 4:**
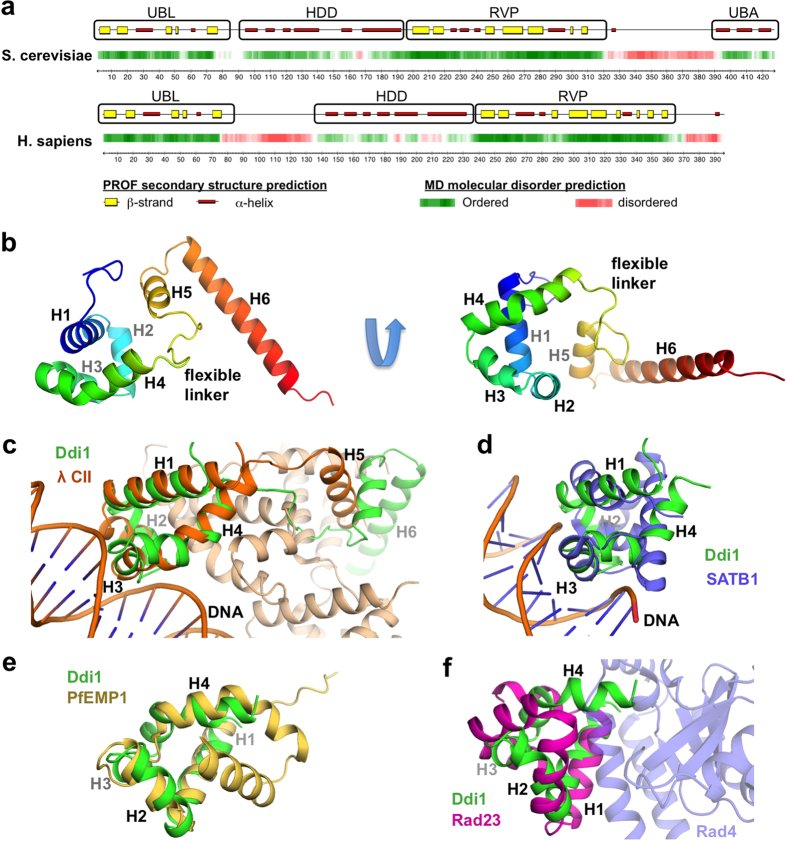
Ddi1 contains two helical domains preceding the protease domain. (**a**) Secondary structure and molecular disorder predictions of the yeast (top) and human (bottom) Ddi1 proteins. Predictions were performed on the PredictProtein server (http://www.predictprotein.org). (**b**) Cartoon of a representative model from the ensemble of HDD solution NMR structures, colored progressively from blue (N-terminus) to red (C-terminus). A flexible linker between helices 4–5 connects the two domains. (**c–f**) Structural superposition of the Ddi1 HDD N-terminal domain (green) with various homologous domains: (**c**) the bacteriophage λ CII transcription activator bound to a DNA duplex (orange, PDB 1ZS4). Other protein chains in the λ CII structure are colored in pale orange, showing the 5^th^ helix mediating tetramerization. (**d**) SATB1 CUT domain bound to a DNA duplex (blue, PDB 2O4A). (**e**) Intracellular domain of *Plasmodium falciparum* erythrocyte membrane protein 1 (yellow, PDB 2LKL). (**f**) Rad23 XPCB domain (magenta, PDB 2F4M) bound to Rad4 (pale blue).

In an effort to elucidate the function of Ddi1, we extended our structural studies beyond its RVP and UBL domains. As previously observed^29^, secondary structure prediction of Ddi1 shows that there is an α-helical region juxtaposed to the N-terminus of the protease domain (Fig. 4a). This domain is separated from the UBL domain by a linker of variable length in different organisms, but is always juxtaposed to the RVP domain. To confirm that this region effectively forms one or many folded domains, we expressed ^15^N,^13^C-labeled Ddi1 (residues 86–196) and characterized its structure by NMR. Its ^1^H,^15^N HSQC spectrum showed good signal dispersion in the proton dimension, indicating that the construct is folded (Supplementary Fig. S4a). We confirmed that this region of Ddi1 effectively adopts a folded structure using ^15^N-^1^H heteronuclear NOE, which shows positive values except for the flexible N- and C-termini (Supplementary Fig. S4b). Because this helical region is folded and conserved across Ddi1 orthologs (Supplementary Fig. S4c), we named it the Helical Domain of Ddi1 (HDD).

The solution structure of HDD was determined using dihedral and NOE distance restraints, as well as residual dipolar couplings (RDCs) (Supplementary Table 3). The HDD actually consists of two alpha-helical domains (Fig. 4b): the N-terminal domain (residues 89–141) is a bundle of four helices with a hydrophobic core formed by some of the most conserved residues of the Ddi1 HDD. The C-terminal domain (residues 150–190) forms a hairpin with two helices, with a small hydrophobic core involving helix 1 and the first portion of the long helix 2. The structure calculation converged for each domain, with backbone average pairwise rmsd of 0.57 and 0.99 Å for the N- and C-terminal domains, respectively. However, there is considerable variability in the relative position of each domain (Supplementary Fig. S5a). A 10-residue linker with lower heteronuclear NOE values (Supplementary Fig. S4b) as well as low sequence conservation amongst Ddi1 orthologs (Supplementary Fig. S4c) connects the two parts of HDD. No long-range ^1^H-^1^H NOE was detected between the N- and C-terminal domains, implying that they do not pack against each other. To determine the extent of the dynamic motion between the two domains, we analyzed small-angle X-ray scattering (SAXS) data using ensemble optimization^37^,^38^. The wide size and *R*_*g*_ distributions of the optimized ensembles of tethered domains structures hint to a dynamic regime with a slightly more compact configuration than expected from a random pool of structures (Supplementary Fig. S6). Finally, the alignment tensor rhombicities are significantly different, further confirming they tumble independently from each other (Supplementary Fig. S5b,c).

To gain insight into the potential function of the HDD region of Ddi1, we used the structure as a query to search for homologous domains in the Protein Data Bank (Supplementary Fig. S7a). Overall, we find that the HDD N-terminal domain (HDDnt) is similar to a wide and disparate set of alpha-helical bundle structures. However, the HDDnt displays striking similarity to DNA-binding domains from transcriptional regulators. Notably, the DNA-binding motif of the bacteriophage λ cII transcription activator has a Cα rmsd of 2.9 Å with HDDnt (Fig. 4c). The domain is followed by a flexible tether and a long helix that can take multiple positions^39^, similar to the C-terminal domain of the HDD. This λ cII C-terminal helix mediates tetramer formation upon DNA-binding. The HDDnt domain is also similar to the POU-specific OCT-1 DNA-binding domain (rmsd 3.9 Å), as well as to the homologous CUT domain from human SATB1 (3.7 Å, Fig. 4d). These DNA-binding domains are all four-helical bundles that insert helix 3 into the major groove and recognize specific DNA sequences (Fig. 4c,d). The fold search also revealed similarity between HDDnt and the intracellular domain of the erythrocyte membrane protein 1 from *Plasmodium falciparum*, but the latter is a five-helical bundle, and only the first four helices are similar to the HDDnt, suggesting their function are likely unrelated (Fig. 4e). Finally, weak sequence homology prompted us to compare the structure of HDDnt to helical bundles of the Sti1-like family found in other UBL-UBA proteins such as Rad23 (Supplementary Fig. S7b). This domain binds to Rad4/XPC, a DNA-binding protein implicated in nucleotide excision repair. The first three helices of HDDnt are similar to XPC-binding motif of that Rad23 domain, but the 4^th^ helix adopts a completely different orientation (Fig. 4f), suggesting it is unlikely that Ddi1 binds Rad4/XPC. Thus, the Ddi1 HDD is most similar to DNA-binding domains.

### SAXS analysis suggests a dynamic structure for the Ddi1 dimer in solution

The Ddi1 protein consists of four domains with known structure, but how they are positioned to each other in the full-length protein is unclear. We therefore used SAXS to determine the relative position of the UBL and HDD domains with respect to the RVP dimer. Light scattering (dynamic and multiple-angle) was initially used to characterize and optimize solution scattering conditions for multiple Ddi1 constructs, except for the full-length protein, which formed aggregates that impeded analysis under all conditions tested. Constructs comprising the RVP domain (200–325) all formed dimers, as expected (Supplementary Fig. S8a). Ddi1 86–325 slightly aggregated at pH 7, but increasing the pH and adding glycerol reduced the aggregation.

**Figure 5:**
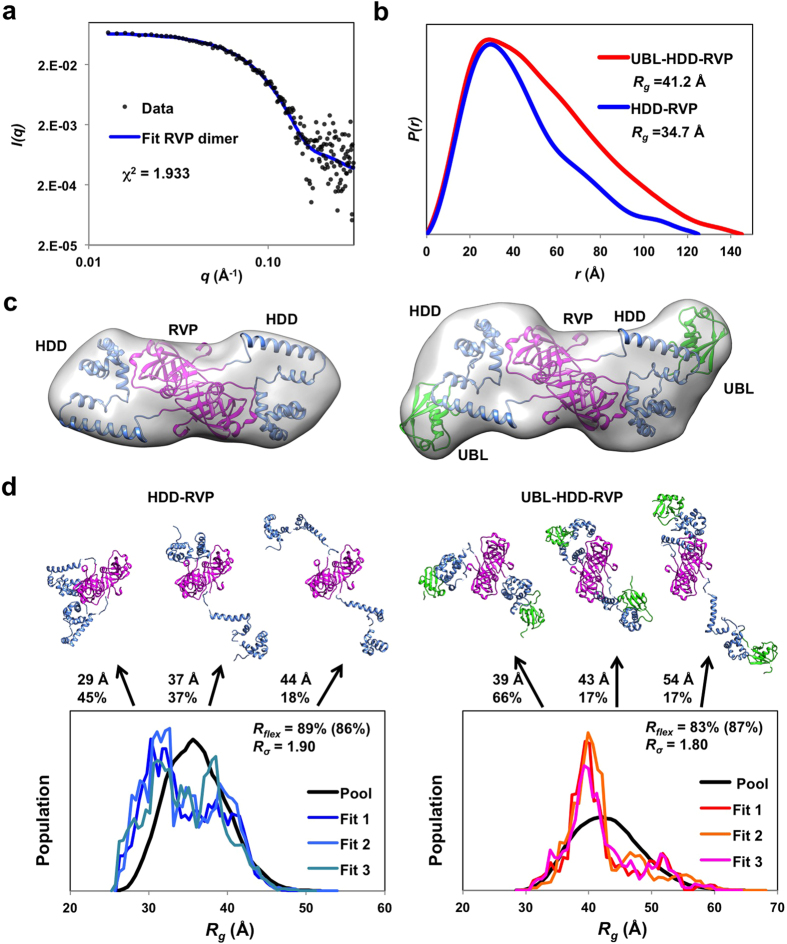
SAXS analysis of the Ddi1 dimer in solution. (**a**) SAXS data (circles) and calculated scattering curves (blue line) derived from the dimeric Ddi1 RVP domain crystal structure (200–325), displayed as a double-logarithmic plot. (**b**) SAXS pair-distance distribution functions for Ddi1 HDD-RVP (86–325) or UBL-HDD-RVP (2–325). (**c**) Modeling of SAXS data for HDD-RVP (left) and UBL-HDD-RVP (right), using the NMR structure of the UBL (green) and HDD (blue) domains, and the crystal structure of the dimeric protease domain (magenta). The surface represents the average of all models generated, contoured at 1.1× the volume of the particle. The cartoon displays a representative model from the ensemble. (**d**) Dynamic ensemble analysis of Ddi1 HDD-RVP (left) and UBL-HDD-RVP (right) using EOM. Each graph shows the distribution of *R*_*g*_ for a pool of 10,000 structures randomly generated, and three sets of 100 ensembles that best fit the data. The structures of the best ensemble is shown on top, with *R*_*g*_ and fraction indicated for each. These models suggest the behaviour of the flexible protein in solution, and does not represent the only solution.

SAXS data from the RVP domain fit well to the dimeric crystal structure reported here, with a χ^2^ of 1.9, confirming that the Ddi1 RVP adopts the same conformation in solution as observed in the crystal structure (Fig. 5a). SAXS data were also acquired on Ddi1 86–325 (HDD-RVP) and 2-325 (UBL-HDD-RVP). The *P(r)* function reveals an asymmetrical pattern characteristic of elongated structures (Fig. 5b). To determine the relative positions of each domain, the SAXS data were fitted with the NMR structure of the UBL, the NMR structure of the HDD domain, and the crystal structure of the RVP domain as inputs. P2 symmetry was imposed, and the RVP domain position was kept constant. SAXS modeling of the Ddi1 HDD-RVP domains yielded two classes of structures, where the HDD domain extends on either side of the protease domain (Fig. 5c and Supplementary Fig. S8b). Calculations with the UBL-HDD-RVP data produced four classes of structures with similar overall shapes (Fig. 5c). The position of the UBL domain is highly variable and can be located on multiple sides of the HDD domain (Supplementary Fig. S8b). To determine the extent of the dynamics and reveal potential interactions among the domains, we also analyzed SAXS data using ensemble optimization^37^,^38^. In this approach, a pool of 10,000 models is generated from the structure of individual domains, connected by flexible linkers, to define the potential conformational space of the multi-domain protein. Then, a genetic algorithm is used to select a subset of conformers that best fit the experimental scattering data. In this case, P2 symmetry was maintained for the core RVP domain, but no symmetry was imposed on the UBL and HDD domains. Excellent fits were obtained for both data sets, and three independent calculations yielded similar *R*_*g*_ and *D*_*max*_ distributions for the best-fit ensemble (Fig. 5d and Supplementary Fig. S9). The ensembles of HDD-RVP structures have more compact structures with smaller *R*_*g*_ values than the pool (Fig. 5d). However analysis of *D*_*max*_ showed no clustering (Supplementary Fig. S9b), and the quantity *R*_*flex*_ is close to that of the pool, suggesting the protein is flexible. Similar results were obtained for the UBL-HDD-RVP construct, albeit with a class of relatively compact structures dominating the ensemble (Fig. 5d). However, an overlay of these compact structures revealed no favored arrangement, and *R*_*flex*_ is also high, reflecting flexibility. Overall, our analysis suggests that the Ddi1 protease dimer is flanked by flexible UBL and HDD domains that are in dynamic exchange in solution, albeit with a tendency towards more compact configurations.

## Discussion

The focus of this study was to investigate the structure of yeast Ddi1 and the interactions mediated by its UBL domain. Surprisingly, we found that the UBL was unable to bind any of the UIMs that we tested, including all four UIMs found in the Ufo1 protein. Similar results were obtained by Nowicka *et al*.^34^. Based on the latter publication, we confirmed that the yeast UBL domain binds to ubiquitin. However, our ITC-based affinity measurements deviate from the reported affinities measured by NMR (150 and 45 μM for the UBA and UBL, respectively^34^), which could be attributed to difference in buffers (sodium phosphate pH 6.8 versus HEPES pH 7.4 here) and/or the use of different constructs. Indeed, the NMR titrations were carried out with isolated domains, as opposed to the ITC titrations that were performed in the context of the full-length dimeric protein. The association rate constants of protein:protein interactions is dependent on translational and rotational diffusion rates and protein electrostatics, which vary with protein size and depends on the context in which the domain is positioned^40^. These factors could explain the 3-fold difference in *K*_*d*_ (about 0.7 kcal/mol in free energy) we measured for the Ddi1 UBA domain. Moreover, the construct used by Nowicka *et al*. lacked the initiator Met1, which alters the position of Asp2, poised to interact with ubiquitin (Fig. 2b). This would reduce the affinity of our construct for ubiquitin, and we indeed observe a 6-fold difference. Whether the yeast Ddi1 protein retains its N-terminal Met1 is unknown, but proteins with a charged residue in the second position are typically not excised, and often acetylated^41^. Nevertheless, our data are overall consistent with yeast Ddi1 having two independent binding sites for ubiquitin. We also found that full-length yeast Ddi1 binds K48-Ub_2_ with a 1:1 stoichiometry. These results could be explained by a mixture of binding modes, including binding to the UBL and UBA within the same Ddi1 molecule, or two UBL or two UBA domains in the dimer. The presence of two ubiquitin-binding domains in yeast Ddi1 may explain why some orthologs lack one or the other domain. For example, the *S. pombe* ortholog Mud1 does not have a UBL domain, but its UBA domain binds tightly to K48-Ub_2_^5^. However, in an accompanying publication^36^, we found that the UBL domain of human Ddi2, which lacks a UBA domain, does not bind to ubiquitin, implying that ubiquitin-binding is not a conserved attribute of the Ddi1-like eukaryotic family of proteins.

Our crystal structure of the RVP domain reveals that the protease domain binds the N-terminus of the protein construct, which adopts an extended β conformation through interaction with a conserved loop adjacent to the active site (Fig. 1). This loop normally forms a flap in aspartyl proteases of retroviruses such as HIV-1, but in our structure, the loop forms an extensive hydrogen-bonding network with the N-terminal segment pseudo-substrate. This conformation is likely a crystallization artefact for the following reasons: in the previous crystal structure of the yeast Ddi1 RVP domain^30^, the segment 180–198 is disordered and not observed in the active site; the construct that was crystallized is not cleaved in solution (Supplementary Fig. S1); the segment 185–191 is actually part of the HDD domain, which adopts an alpha-helical conformation in solution (Fig. 4). Yet, the pseudo-substrate N-terminal segment, adopts a conformation similar to HIV-1 protease peptide substrates. This suggests that the yeast Ddi1 RVP domain indeed functions as a hydrolytic enzyme against polypeptides, and that the observed conformation likely represents a model of substrate binding.

Unlike retroviral aspartyl proteases, which are able to cleave peptide substrates *in vitro*, Ddi1 did not exhibit any protease activity in our proteomic screen against a library of peptides derived from the yeast proteome (Supplementary Fig. S2). While this could mean that this domain might simply not be a protease, it could also be that the protease is activated only in the context of its interactions with another protein. The newly identified HDD domain could serve as a substrate anchor in this context (Fig. 4). Our SAXS data show that the HDD extends on either side of the RVP dimer and could thus serve as a landing platform for a substrate (Fig. 5c). We report a similar configuration in human Ddi2^36^ where the HDD is juxtaposed to the protease dimer, suggesting a conserved functional relationship between the HDD and RVP domains. However, ensemble modeling of the SAXS data shows that the UBL and HDD domains are likely dynamic and can adopt multiple configurations (Fig. 5d). Considering the underdetermined nature of SAXS data, we cannot conclude whether the UBL-HDD-RVP module adopts a single rigid or multiple dynamic conformations. However, we note that NMR titrations with the UBL and the HDD-RVP constructs revealed no interaction, in agreement with the dynamic nature of the UBL-HDD-RVP module.

The role of the HDD remains unclear, but it is likely mediating interactions with a potential substrate of the Ddi1 protease. Sti1-like domains homologous to the HDD have indeed been implicated in protein-protein interactions. In particular, the Rad23 Sti1-like domain forms a complex with the nucleotide excision repair protein Rad4/XPC^42^,^43^, and the role of this interaction might be to protect Rad4/XPC from proteasomal degradation^44^. Dsk2/ubiquilin also contains a Sti1-like domain that has been proposed to bind the Hsp70-like protein Stch^45^. More strikingly, the structural homology with DNA-binding domains hints to the possibility that Ddi1 might be recognizing specific DNA motifs itself. The domain has some hallmarks of DNA-binding domains, such as a conserved basic residue (Arg131) that faces the DNA phosphate backbone in the structure alignment with the bacteriophage λ CII transcription activator bound to a DNA duplex. This is consistent with the observation that Ddi1 localizes to the nucleus^24^. As Ddi1 is implicated in the DNA-damage response and cell cycle checkpoints^11^,^15^, it is possible that Ddi1 HDD binds to DNA damage sites whose regulation requires an ubiquitin-dependent proteolysis event. Future work should focus on identifying binding partners for Ddi1 HDD.

## Methods

### Protein expression and purification

The DDI1 gene was amplified by PCR from yeast genomic DNA and used as a template to generate fragments containing UBL (residues 2–80), HDD (86–196), RVP (185–325), HDD-RVP (86–325), and UBL-HDD-RVP (2–325). Yeast Ufo1 UIM1-4 (residues 512–668), UIM1 (512–539), UIM2 (542–569), and UIM3 (577–608) were similarly amplified by PCR. Rpn10 cloning was described previously^46^. These fragments were cloned into the pGEX-6p1 plasmid in-frame with an N-terminal GST tag *via Bam*H1 and *Xho*I sites. Constructs were expressed overnight with 0.5 mM IPTG at 20 °C in *E. coli* BL21(DE3) cells, resuspended in TBS buffer (50 mM Tris-HCl, pH 8.0, 100 mM NaCl, 5 mM β-mercaptoethanol) supplemented with 1 mM EDTA, and lysed by sonication. The fusion protein was purified using glutathione-sepharose affinity and eluted with 20 mM glutathione dissolved in TBS. The fusion protein was cleaved overnight at 4 °C with the 3C protease and applied onto size-exclusion Superdex 200 or Superdex 75 16/600 chromatography columns (GE Healthcare). Contaminant GST was removed using glutathione-sepharose resin. Gel filtration was performed in NMR buffer (10 mM HEPES-NaOH, pH 7.0, 50 mM NaCl, 5 mM β-mercaptoethanol) for Ddi1 UBL, HDD, HDD-RVP, and UBA and Ufo1 UIM1-4, or SAXS buffer (10 mM Tris-HCl, pH 8.0, 50 mM NaCl, 5% glycerol, 5 mM β-mercaptoethanol) for constructs 2–325, 86–325, 185–325, and 86–196. Ufo1 UIM4 (651–668) was synthesized chemically. Single UIMs were further purified by C18 reverse chromatography and lyophilized prior to resuspension in NMR buffer.

Full-length yeast Ddi1, UBL (1–80), UBL-RVP (1–325), and RVP-UBA (180–428) were also cloned into pET16b vector (Novagen) in-frame with an N-terminal histidine tag and used for ITC measurements. They were expressed in *E. coli* BL21(DE3)RIL host cells; subsequently resuspended in 50 mM Tris-HCl, pH 8.0, 50 mM NaCl, and 1 mM EDTA; and lysed by three passages through an EmulsiFlex-C3 high pressure homogenizer (Avestin, Canada) at 1200 bar. Proteins were purified using nickel affinity chromatography and eluted with 250 mM imidazole. Afterwards, they were dialyzed overnight into 50 mM HEPES, pH 7.4, 150 mM NaCl, and 10% glycerol and further purified by size-exclusion chromatography on a Superdex 200 16/60 gel filtration column (GE Healthcare). Individual fractions were analyzed by SDS-PAGE and/or Western blot.

The Ddi1 UBL with an N-terminal His-tag (residues 1–80) was expressed as ^15^N- and ^15^N/^13^C-labeled proteins in cells grown in minimal medium containing 0.8 g/L [^15^N] ammonium chloride and 2 g/L d-[^13^C] glucose, as required. The purification procedure was the same as described above.

K48-linked Ub_2_ was synthesized using K48C and D77 ubiquitin mutants mixed with human E1 and yeast Cdc34, as previously described^46^,^47^. The products were purified by cation-exchange chromatography (mono S 5/50 GL, GE Healthcare) using 25 mM sodium acetate, pH 4.5, and 1 M NaCl for elution, and buffer-exchanged in ITC buffer.

### X-ray crystallography

Ddi1 RVP (residues 185–325) was purified by gel filtration and concentrated to 8 mg/mL in 10 mM Tris-HCl, pH 7.4, 50 mM NaCl and 1 mM DTT. MALDI-TOF analysis revealed a single species with an average molecular weight of 16,320 Da (predicted 16,313 Da). The protein was crystallized by vapor diffusion using the sitting drop technique by mixing 1 μL of protein solution with 1 μL of crystallization solution (0.1 M phosphate-citrate, pH 4.2, 0.4 M NaCl, 20% PEG 8000). A crystal grew in 1–2 days. The crystal was cryo-protected by addition of 15% glycerol to the crystallization solution.

X-ray diffraction data at 100K were acquired at the CHESS beamline A1a (Supplementary Table 1). A total of 240 images with an oscillation angle of 0.5 were collected. Reflections were integrated using iMOSFLM and scaled with SCALA as implemented in the *CCP4* package^48^. The structure was determined by molecular replacement with the program Phaser^49^, using chains A and B of the yeast Ddi1 RVP structure as a search model (PDB code 2I1A^30^). Model building was performed using the program COOT^50^. Restrained and TLS refinement were performed using Refmac5^48^.

### Nuclear magnetic resonance spectroscopy

NMR spectra were acquired from 350 μl samples of 0.2 mM ^15^N-labeled Ddi1 UBL for binding site mapping or 0.5 mM ^13^C/^15^N-labeled Ddi1 UBL for structural determination in a 50 mM sodium phosphate buffer, pH 7.4, containing 0.5% glycerol and 5% D_2_O/95% H_2_O. All NMR data for the UBL were collected at 25 °C on a 600 MHz Bruker Avance spectrometer equipped with a triple resonance (^15^N/^13^C/^1^H) cryoprobe. For determination of the sequence-specific resonance assignments for the UBL domain, a series of double and triple resonance spectra were collected as described previously^51^,^52^. ^1^H-^1^H distance constraints required to calculate the structure were derived from NOEs identified in 3D ^15^N/^1^H NOESY-HSQC, and ^13^C/^1^H HSQC-NOESY spectra, which were acquired with an NOE mixing time of 120 ms. Specific interaction of proteins and peptides with the Ddi1 UBL was monitored by changes induced in the positions of signals of ^15^N-labeled Ddi1 UBL 2D ^15^N/^1^H HSQC spectra using a recently described combined minimal shift approach^53^. A two-fold molar excess (0.4 mM) of Rpn10, Ddi1 HDD-RVP, Ddi1 UBA, Ufo1 UIM1-4, UIM1, UIM2, and UIM4 was added in these experiments. All spectra were processed using Topspin 3.2 (Bruker) and analyzed using Sparky (www.cgl.ucsf.edu/home/sparky).

All data sets for the Ddi1 86–196 HDD domain were acquired in HEPES-based NMR buffer at 30 °C on a 600 or 850 MHz Bruker NMR spectrometer both equipped with a triple-resonance (^1^H, ^13^C, ^15^N) cryoprobe. Heteronuclear ^1^H-^15^N NOE values were measured at 600 MHz, as described^54^. ^15^N-^1^H residual dipolar couplings were measured in 10 mg/mL Pf1 bacteriophage^55^ at 600 MHz using a sensitivity-enhanced HSQC-IPAP experiment^56^. Backbone assignments were performed on ^15^N,^13^C-labeled protein samples (0.5 mM) using CBCACONH and HNCACB NMR experiments. ^1^H-^1^H distance constraints required to calculate the structure were derived from NOEs identified in 3D ^15^N/^1^H NOESY-HSQC, and ^13^C/^1^H HSQC-NOESY spectra, which were acquired at 850 MHz with an NOE mixing time of 120 ms.

The family of converged structures for Ddi1 UBL and HDD were initially calculated using Cyana 2.1^57^,^58^. NOE-derived restraints from 3D ^15^N- and ^13^C-edited NOESY spectra, which were assigned using combined automated NOE assignment and structure determination protocol, were used to produce preliminary structures. Backbone torsion angle constrains were generated from assigned chemical shifts using the program TALOS+^59^. For the UBL, hydrogen bond constraints involving residues with slowly exchanging amide protons were used in the calculations. Subsequently, five cycles of simulated annealing combined with redundant dihedral angle constrains were performed to produce a set of 43 converged structures with the lowest Cyana target function, no distance constraint violation and van der Waals violations greater than 0.2 Å, and no dihedral angle constraint violation greater than 5°. These were further refined in explicit solvent using the YASARA forcefield^60^. The structure of the HDD domain was further refined in XPLOR-NIH to incorporate residual dipolar couplings for residues displaying heteronuclear NOE values above 0.6, i.e. 90–141 (N-terminal domain) and 151–187 (C-terminal domain). Initial estimates and Monte Carlo calculations of the alignment tensor *D*_*a*_ and *D*_*r*_ were obtained using the software MODULE^61^. Satisfactory *R*_*dip*_ values of 30% and 35% were obtained for the N- and C-terminal domains, respectively, prior to the RDC refinement in XPLOR-NIH, indicating the accuracy of the NOE-derived structure. *D*_*a*_ and *D*_*r*_ were then optimized using a grid-search in XPLOR-NIH. Structures with the lowest total energy were selected.

### Small-angle X-ray scattering (SAXS)

Small-angle X-ray scattering data sets were collected on an in-house Anton Paar SAXSess camera equipped with a PANalytical PW3830 X-ray generator and a Roper/Princeton CCD detector. The beam length was set to 18 mm, and the beam profile was recorded using an image plate for subsequent desmearing. Scattering data were collected at 4 °C at protein concentrations of 4.0 and 8.0 mg/mL for 1 hour for Ddi1 185–325, 5.0 and 10.0 mg/mL for 2 hours for 2–325, 5.0 and 10.0 mg/mL for 2 hours for 86–325, and 5.0 and 10.0 mg/mL for 2 hours for 86–196. Background scattering from the SAXS buffer was measured for 2 hours. Dark current correction, scaling, buffer subtraction, binning, desmearing, and merging were performed using SAXSquant 3.0 (Anton Paar). The merged scattering curves were then analyzed with different software included in the ATSAS package^62^. Scattering data were fitted to chains A & B of the Ddi1 RVP crystal structure using CRYSOL, pair-distance distributions and *R*_*g*_ values were calculated using GNOM. Molecular weights were estimated using the *Q*_*r*_ invariant as described^63^. Ensemble optimization of the Ddi1 86–196 structure against the SAXS data was performed using EOM 2.0 by generating 10,000 structures with the NMR structures of the N-terminal (a.a. 89–141) and C-terminal (a.a. 150–191) HDD domains, selected using a genetic algorithm. Modeling of data collected from Ddi1 2–325 and 86–325 was performed using CORAL with the NMR structure of the UBL (a.a. 2–75), the two HDD domains (a.a. 89–141 & 150–191) and the dimeric crystal structure of the RVP (a.a. 200–325). Twenty models with χ^2^ < 1.6 were generated and averaged using DAMAVER, with average χ^2^ of 1.36 and 1.19 for HDD-RVP and UBL-HDD-RVP, respectively. The resulting coordinates were used to generate pseudo-densities using Situs-pdb2vol^64^ and contoured 10% above the particle volume derived from the Porod invariant (109,000 and 148,000 Å^3^ for HDD-RVP and UBL-HDD-RVP, respectively) using UCSF-Chimera^65^. EOM 2.0 was used to generate 10,000 structures using the same domains as used in CORAL, with P2 symmetry imposed only on the RVP domain, using the genetic algorithm for conformer selection. The genetic algorithm was performed 100 times thrice to estimate the variability in the distribution of *D*_*max*_ and *R*_*g*_ values.

### Isothermal titration calorimetry (ITC)

All calorimetric titrations of ubiquitin with full-length yeast Ddi1 and truncated variants were performed in 50 mM HEPES, pH 7.4, 150 mM NaCl at 25 °C using a VP-ITC system (MicroCal, GE Healthcare Life Sciences). For full-length Ddi1, 9 μL aliquots of 1.42 mM bovine ubiquitin (Sigma, cat. no. U6253) were injected stepwise into a sample cell containing 1.43 ml of 97 μM Ddi1 protein (concentration calculated to monomer). For UBL-RVP, 9 μl aliquots of 2 mM bovine ubiquitin were injected stepwise into a sample cell containing 1.43 ml of 133.1 μM Ddi1 UBL-RVP protein, and for RVP-UBA, 9 μl aliquots of 796 μM bovine ubiquitin were injected stepwise into a sample cell containing 1.43 ml of 64.8 μM Ddi1 RVP-UBA protein. The control dilution experiment, in which ubiquitin was injected into buffer alone, was also performed. All proteins used for titrations were properly dialyzed against buffer at 4 °C overnight, and their exact concentrations were determined by HPLC amino acid analysis. Titration of K48-Ub_2_ with full-length yeast Ddi1 was performed in 50 mM HEPES, pH 7.4, 150 mM NaCl at 25 °C. Nine-microliter aliquots of 833.5 μM K48-Ub_2_ were injected stepwise into a sample cell containing 1.43 ml of 48.5 μM Ddi1 protein. Data sets were analyzed using Origin, using a one-site model by varying *N*, *K*_*d*_ and Δ*H*. For the titration with Ddi1 FL and ubiquitin, the data were also fitted to a two-sites model, where *N* was fixed to 1.0 and *K*_*d*_ and Δ*H* were floating variables for both sites. The range of values was determined by allowing the χ^2^ value to increase up to 37.3, observed at *K*_*d1*_ = 50 μM and *K*_*d2*_ = 926 μM, which still gives a satisfactory fit. The minimum χ^2^ value of 21.4 was observed at *K*_*d1*_ = 175 μM and *K*_*d2*_ = 575 μM.

### PICS assay and analysis

The PICS procedure was carried out as previously described^31^, and further details are included in our back-to-back publication^34^. Briefly, the amine-protected yeast proteome-derived peptide library (1 mg/ml) was incubated in 200 μL buffer with 4 μg of full-length yeast Ddi1 WT. The reaction was incubated for 12 h at 37 °C. The proteolytic cleavage assays were carried out in of 100 mM sodium acetate, 300 mM NaCl, pH 4.0, 100 mM sodium acetate, 300 mM NaCl, pH 5.0, or 100 mM HEPES, 300 mM NaCl, pH 7.0. As negative controls, we used full-length Ddi1 with an inactivating mutation in its catalytic site (D220A), as well as a mock reaction with buffer. As a positive control, we tested the HIV-1 protease cleavage profile in 100 mM Na acetate, 300 mM NaCl, pH 4.7, using wild-type and the catalytically inactive D25N mutant in a 1:200 protease-to-library ratio.

Data were analyzed by a series of predesigned queries in Microsoft Access database management software. First, lists of identified peptides from each MS run were filtered for peptides containing products of N-terminal modification by biotinylation. Second, peptides with over 80% confidence were picked for the tested enzyme, while peptides with over 10% confidence were picked for control reactions. To properly subtract the background signal, the list of peptides identified in the tested enzyme reaction was screened for peptides present in the mock reaction as well as in the reaction with catalytically inactive, and those peptides were removed from processing. Finally, peptides identified in the original unprocessed peptide library were removed from the analysis.

The final cleared list of identified peptides was then aligned with a FASTA proteomics database used for proteomics database search to determine the N-terminal portions of cleaved peptides. If multiple computationally identified preceding sequences were found for one MS identified peptide, they were removed from processing, while the MS identified peptide sequences were kept in the list for downstream analysis. The final list of substrate peptides containing sequences of five P’ amino acids identified in the MS experiment and five P amino acids identified computationally was then created. The frequency of each amino acid in each particular position was calculated and plotted, yielding the substrate specificity matrix.

## Additional Information

**Accession Codes**: Coordinates and structure factors for the RVP crystal structure were deposited in the PDB under accession code 4Z2Z. Coordinates and restraints for the Ddi1 UBL and HDD solution NMR structures were deposited in the PDB under accession codes 2N7E (BMRB code 25803) and 5KES (BMRB code 30102), respectively.

**How to cite this article**: Trempe, J.-F. *et al*. Structural studies of the yeast DNA damage-inducible protein Ddi1 reveal domain architecture of this eukaryotic protein family. *Sci. Rep*. **6**, 33671; doi: 10.1038/srep33671 (2016).

## Supplementary Material

Supplementary Information
