# Supplementary material for: Structural studies of the yeast DNA damage-inducible protein Ddi1 reveal domain architecture of this eukaryotic protein family
Source: Sci Rep. 2016 Sep 20;6:33671. doi: 10.1038/srep33671 (PMC5028754; doi:10.1038/srep33671)
Supplement: Supplementary Information [file srep33671-s1.pdf]

Supplementary information

Structural studies of yeast DNA damage-inducible protein Ddi1 reveal domain architecture of this eukaryotic protein family

Authors: Jean-François Trempe[1,2,*], Klára Grantz Šašková[3,4], Monika Sivá[3,4,5], Colin Ratcliffe[1], Václav Veverka[3], Annabelle Hoegl[1], Marie Ménade[1], Xin Feng[1], Solomon Shenker[1], Michal Svoboda[3,6], Milan Kožíšek[3], Jan Konvalinka[3,4], Kalle Gehring[1]

[1]Groupe de Recherche Axé sur la Structure des Protéines, Department of Biochemistry, McGill University, Montreal, QC, H3G 1Y6, Canada

[2]Current affiliation: Department of Pharmacology & Therapeutics, McGill University

[3]Gilead Sciences and IOCB Research Center, Institute of Organic Chemistry and Biochemistry of the Academy of Sciences of the Czech Republic, Flemingovo n. 2, 166 10 Prague 6, Czech Republic

[4]Department of Biochemistry, Faculty of Science, Charles University, Hlavova 8, 120 00 Prague 2, Czech Republic

[5]First Faculty of Medicine, Charles University in Prague, Katerinska 32, 121 08, Prague 2, Czech Republic

[6]Department of Physical and Macromolecular Chemistry, Faculty of Science, Charles University, Hlavova 8, 120 00 Prague 2, Czech Republic

*Correspondence: jeanfrancois.trempe@mcgill.ca

Keywords: Ddi1, protease, SAXS, Sti1, UBL, UBA, ubiquitin, Lys48, crystal structure

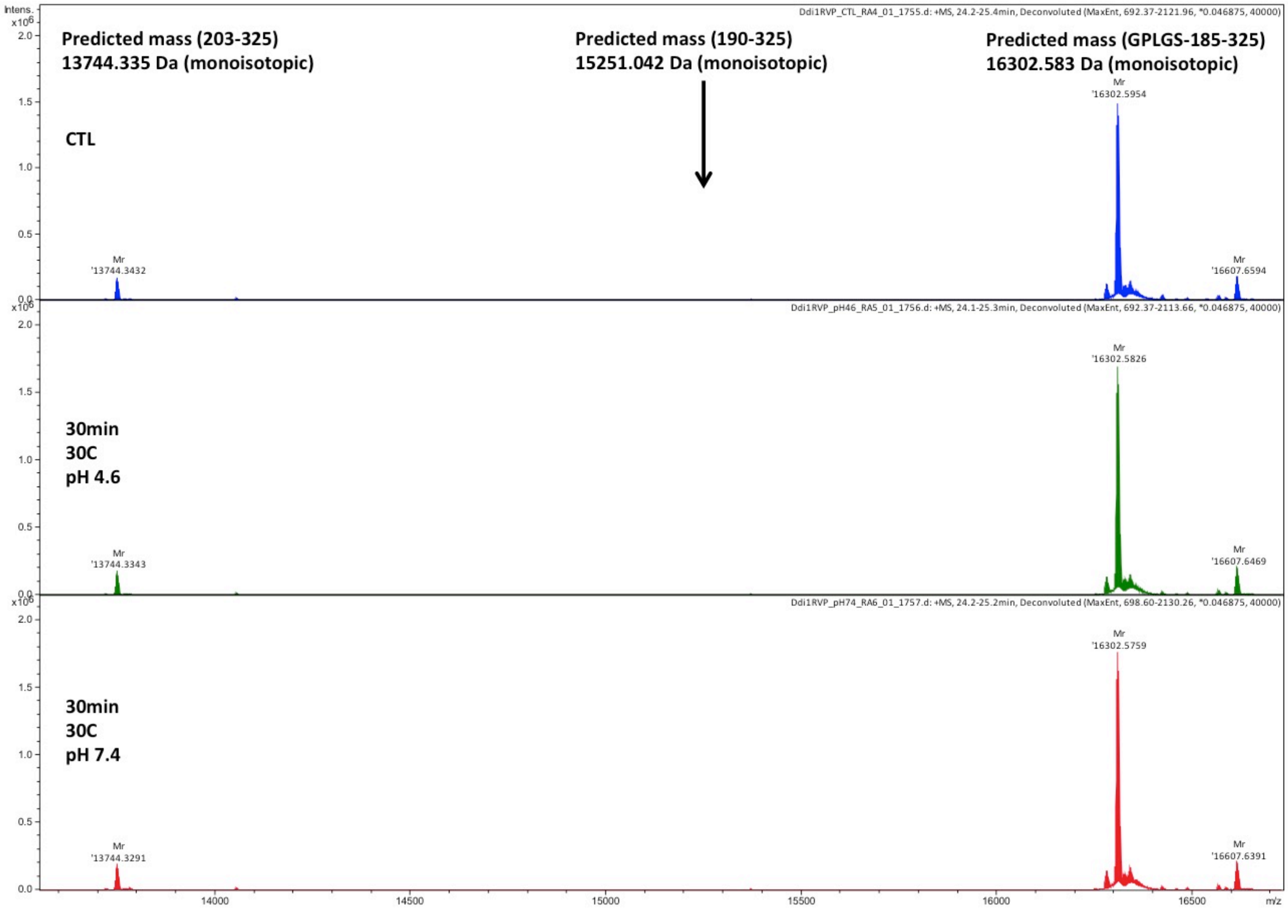

Supplementary Figure S1. The RVP domain of Ddi1 does not auto-proteolyze under neutral or acidic pH. Ddi1 (185-325) protein stock used for crystallization was diluted to 1 mg/mL in 50 mM sodium acetate pH 4.6 or 50 mM HEPES pH 7.4 and incubated 30 min at 30°C. The sample was diluted at 0.1 mg/mL in 0.05% TFA/2% acetonitrile and 20 µL was injected on a Dionex C4 Acclaim 1.0/15mm column followed by a 10 min 4-50% gradient of acetonitrile in 0.1% formic acid, with a flow rate of 40 µL/min. The eluate was analyzed on a Bruker Impact II Q-TOF mass spectrometer equipped with an Apollo II ion funnel ESI source. The multiply charged ions were deconvoluted at 40,000 resolution to yield the isotopically-resolved mass spectra shown above. Peak assignment was performed using the SNAP algorithm, which takes into account natural abundance isotope distribution to assign monoisotopic mass. The main peak in all spectra is within 0.01 Da of the predicted mass. The cleavage product predicted from the crystal structure (190-325, 15251 Da) is absent in all conditions, suggesting it is a crystallization artefact. The contaminant peak observed at 13744 Da corresponds to residues 203-325, where the entire flexible N-terminus has been digested by contaminant proteases. The peak at 16607 Da is an adduct of glutathione (306 Da), used as an eluent of the precursor GST-fusion protein.

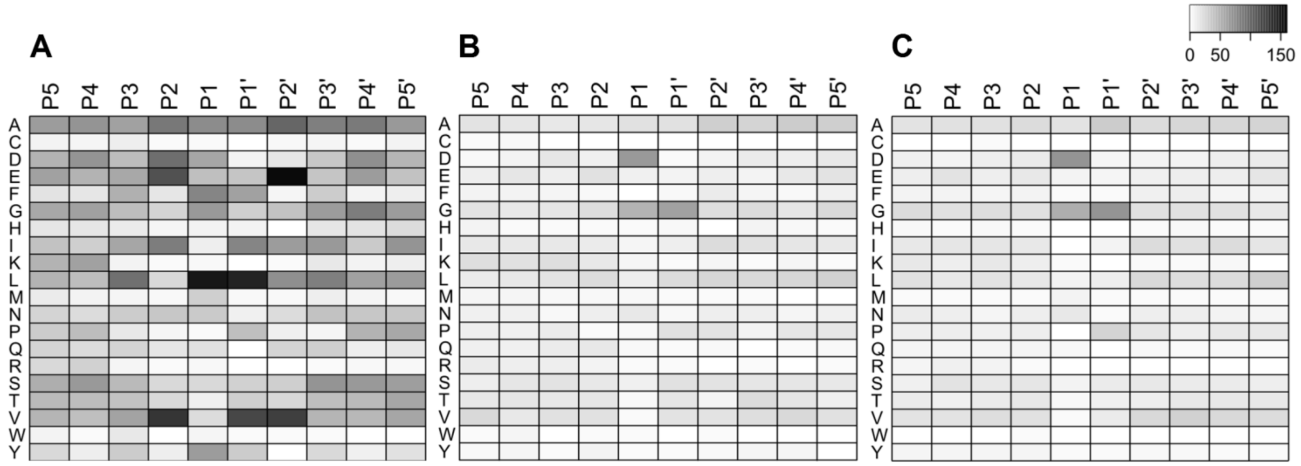

Supplementary Figure S2: PICS (Proteomic Identification of protease Substrates) experiment results. Heat map representation of protease substrate profile demonstrating total counts of given amino acid (described in single letter code) per particular position (P5–P5´) in peptidic substrate on *Saccharomyces cerevisiae* derived peptide library. (**A**) library cleaved by HIV-1 protease (positive control) (**B**) Library cleaved by yeast Ddi1 protein in acetate pH = 4.0 (proteolytic activity assay) (**C**) background profile – uncleaved library (negative control). Color key common for all three heat maps.

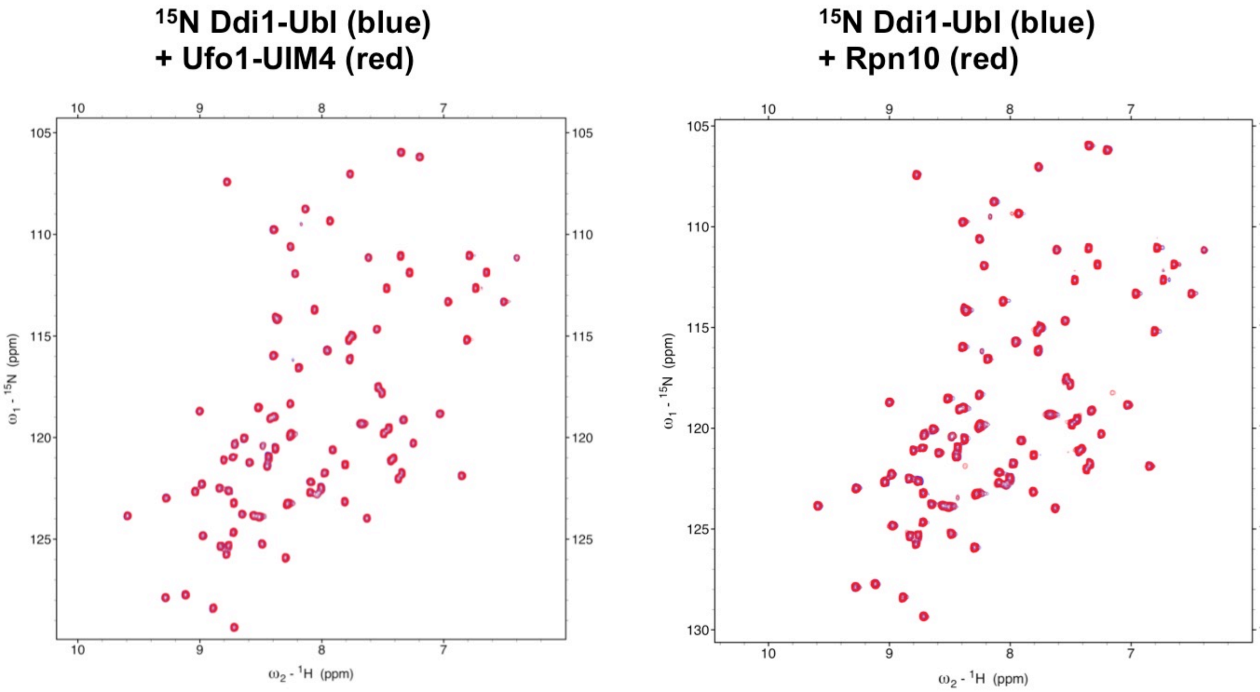

Supplementary Figure S3. The UBL domain of Ddi1 does not bind ubiquitin-interacting motifs. ^{15}N-^{1}H HSQC spectra of the Ddi1 UBL in the presence of Ufo1-UIM4 (left) and Rpn10 (right). The absence of chemical shift perturbation indicates no interaction.

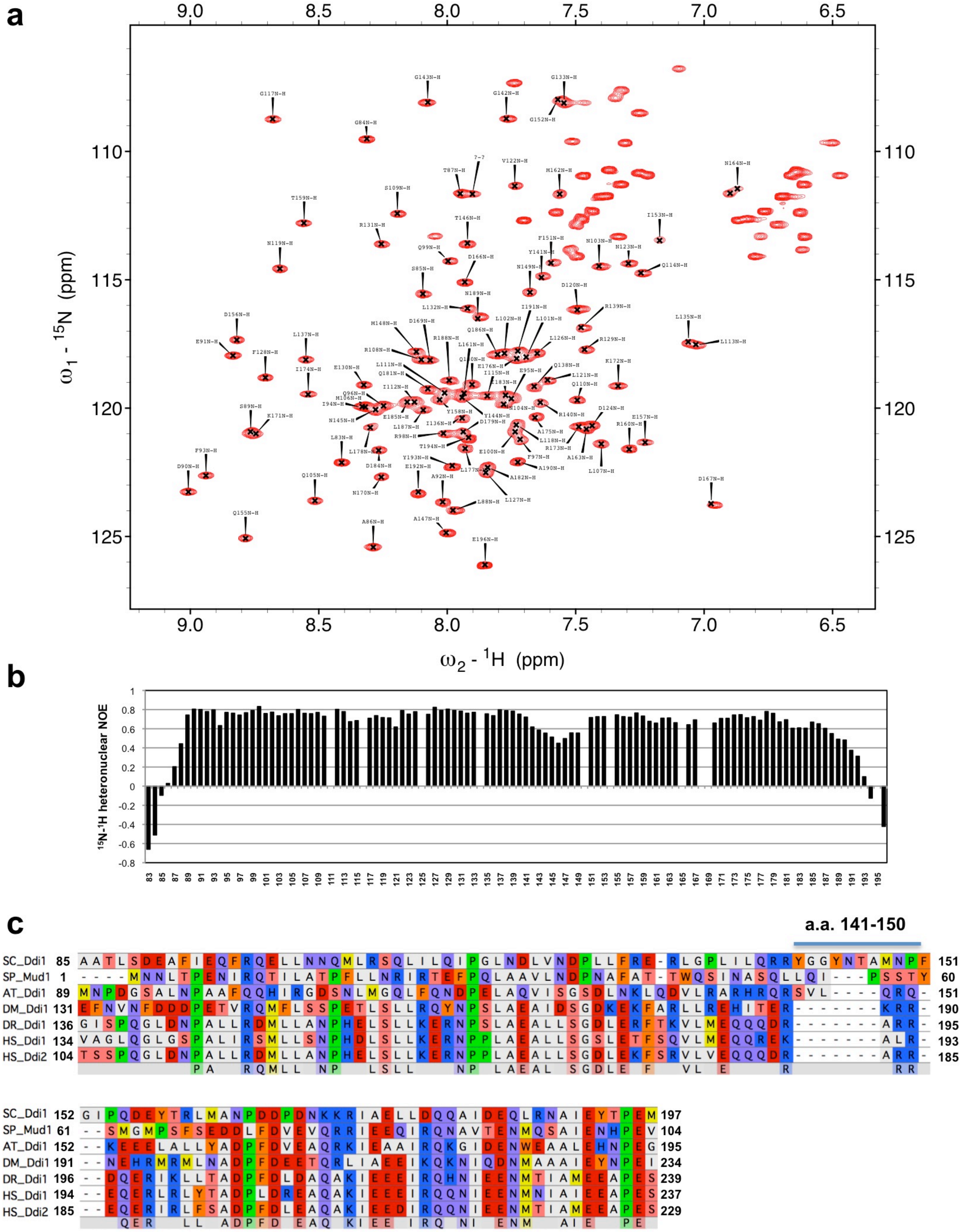

Supplementary Figure S4. NMR characterization of the Ddi1 HDD domain. **(a)** ^{1}H-^{15}N HSQC spectrum of Ddi1 86-196 (HDD), showing a good dispersion of signals in the proton dimension. **(b)** ^{1}H-^{15}N heteronuclear NOEs as a function of residue number. **(c)** Sequence alignment of Ddi1 HDD from different species. SC, *Saccharomyces cerevisiae*; SP, *Schizosaccharomyces pombe*; AT, *Arabidopsis thaliana*; DM, *Drosophila melanogaster*; DR, *Danio rerio*; HS, *Homo sapiens*. The position of the linker tethering the N- and C-terminal domains is indicated.

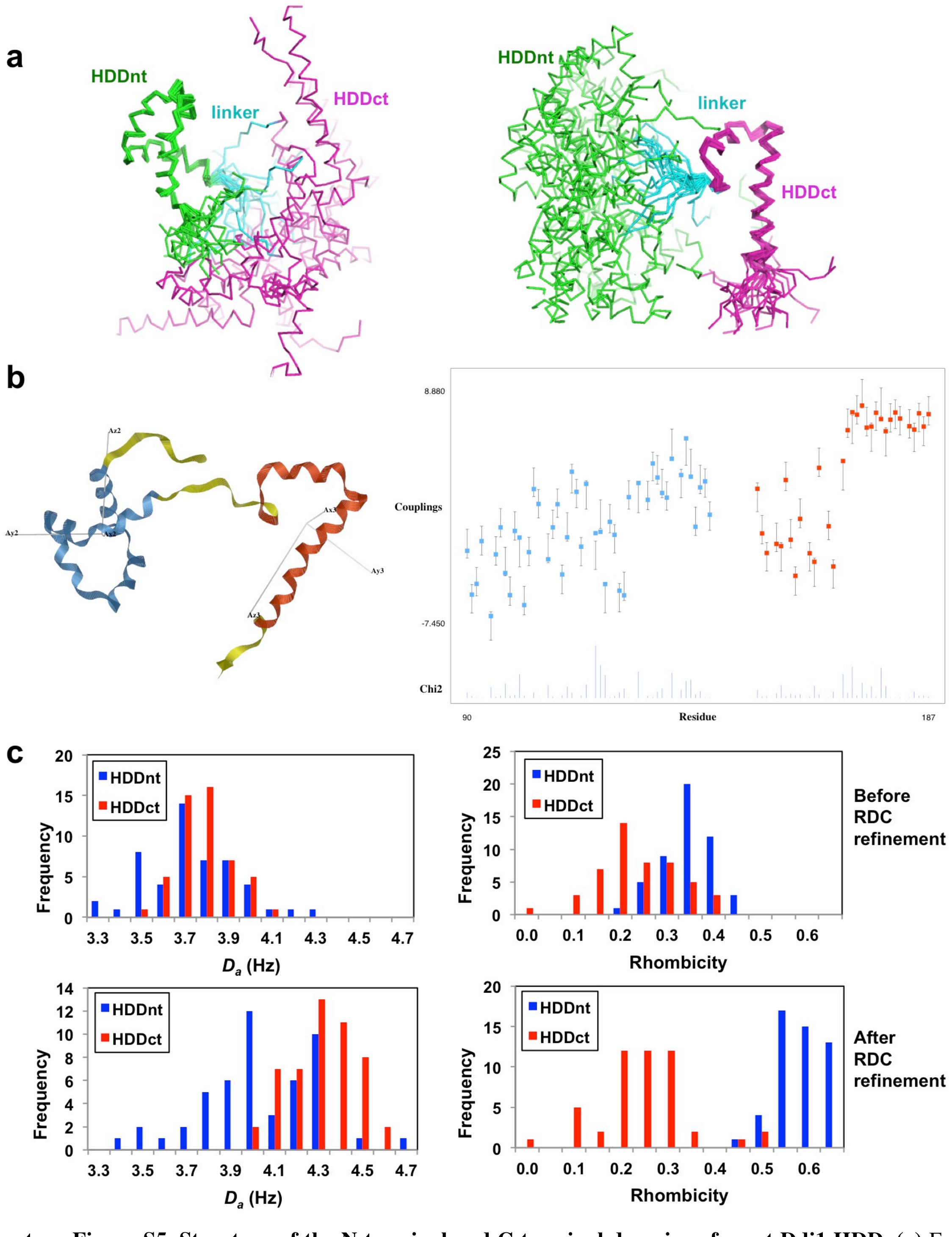

Supplementary Figure S5. Structure of the N-terminal and C-terminal domains of yeast Ddi1 HDD. (a) Ensemble of the solution structure of the yeast Ddi1 HDD**,** superposed on the N-terminal domain (left, green) or C-terminal domain (right, magenta). The linker is shown in cyan. **(b)** ^{15}N-^{1}H RDC tensor analysis of the HDD structure using MODULE. Left, orientation of the order tensors for the two domains of HDD. Right, back-calculated RDC values (blue and red dots) compared with the experimental data (vertical error bars, each 1.0 Hz). The bars at the bottom indicate the Chi^2 deviation for each coupling. **(c)** Monte Carlo simulation to determine the uncertainty on the magnitude (D_a) and rhombicity of the RDC alignment tensors for the two domains. 50 simulations were performed using an uncertainty of ± 0.5 Hz, using the lowest-energy structure calculated before (top) and after (bottom) refinement with RDC.

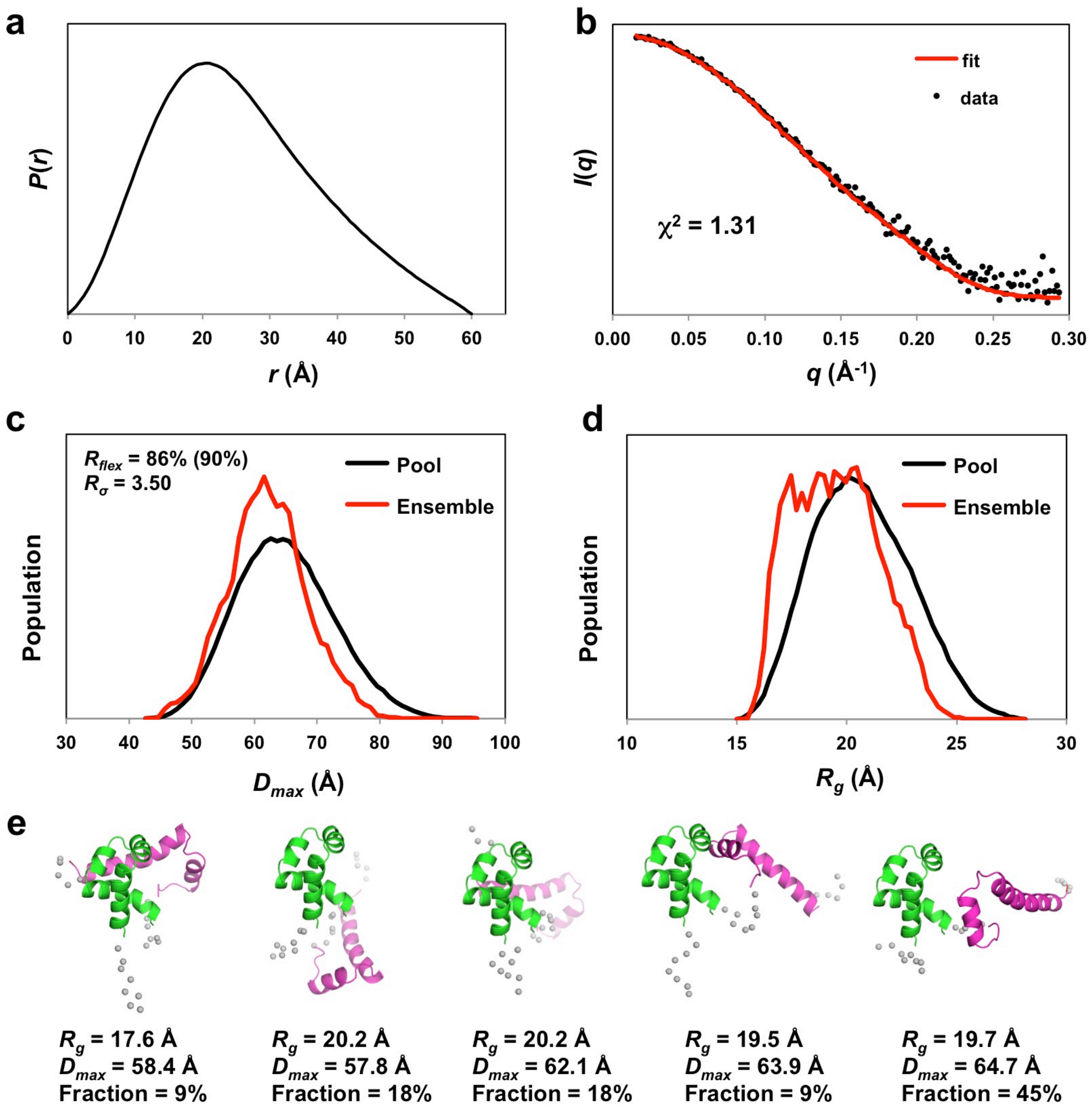

f

R_g (SAXS)	R_h (QELS)	M.wt (SAXS)	M.wt. (MALS)	Calc. M.wt
19.7 ± 0.2 Å	23 ± 2 Å	17 ± 2 kDa	13 ± 1 kDa	13.7 kDa

Supplementary Figure S6. SAXS analysis of the Ddi1 HDD domain. **(a)** SAXS pair-distance distribution function for the HDD domain. The apparent D_{max} is 60 Å. **(b)** Fit of ensemble of EOM-generated structures to experimental data. **(c)** Population distribution of D_{max} for a pool of random structures of the N-terminal and C-terminal domains of HDD (black), compared with an ensemble that best fit the experimental data (red). The ensemble and pool averages are 62.1 and 64.9 Å, respectively. **(d)** Same as in (c), except for the radius of gyration R_g. The ensemble and pool averages are 19.5 and 20.7 Å, respectively. **(e)** Models from the best ensemble solution from the EOM calculation, with R_g, D_{max} and fraction for each model indicated. **(f)** Summary of light (R_h, M.Wt) and small-angle X-ray (Rg, M.Wt) scattering parameters for Ddi1 HDD.

a

##	Scoring Q	Scoring P	Scoring Z	RMSD	N_{align}	N_g	$\%_{seq}$	Query $\%_{sse}$	Match	$\%_{sse}$	N_{res}	×	Target (PDB entry) Title
1	0.31	0.3	3.6	2.22	45	3	11	60	2lkl:A	60	81	☑	STRUCTURE OF THE CORE INTRACELLULAR DOMAIN OF PFEMP1
2	0.29	0.0	1.8	3.64	50	4	16	60	2mqk:A	75	65	☑	SOLUTION STRUCTURE OF N TERMINAL DOMAIN OF THE MUB AAA+ ATPASE
3	0.25	0.1	2.9	2.84	45	2	2	60	1zs4:A	60	82	☑	STRUCTURE OF BACTERIOPHAGE LAMBDA CII PROTEIN IN COMPLEX WITH DNA
4	0.24	0.1	2.7	2.98	45	3	2	60	1zs4:C	60	79	☑	STRUCTURE OF BACTERIOPHAGE LAMBDA CII PROTEIN IN COMPLEX WITH DNA
5	0.24	0.0	0.9	3.86	49	7	12	60	1pou:A	75	71	☑	THE SOLUTION STRUCTURE OF THE OCT-1 POU-SPECIFIC DOMAIN REVEALS A STRIKING SIMILARITY TO THE BACTERIOPHAGE LAMBDA REPRESSOR DNA-BINDING DOMAIN
6	0.23	0.0	1.7	3.70	49	6	6	60	2lvk:A	60	77	☑	SOLUTION STRUCTURE OF CA-BOUND PHL P 7
7	0.23	0.0	2.1	3.45	45	4	9	60	2eo2:A	100	71	☑	SOLUTION STRUCTURE OF THE INSERTION REGION (510-573) OF FTHFS DOMAIN FROM MOUSE METHYLENETETRAHYDROFOLATE DEHYDROGENASE (NADP+ DEPENDENT) 1-LIKE PROTEIN
8	0.23	0.0	2.6	3.63	50	7	8	80	1wmg:B	67	83	☑	CRYSTAL STRUCTURE OF THE UNC5H2 DEATH DOMAIN
9	0.23	0.0	1.5	3.27	45	3	2	60	2avu:C	60	76	☑	STRUCTURE OF THE ESCHERICHIA COLI FLHDC COMPLEX, A PROKARYOTIC HETEROMERIC REGULATOR OF TRANSCRIPTION
10	0.23	0.0	1.3	4.31	51	4	20	60	4yg7:C	60	71	☑	STRUCTURE OF FL AUTOREPRESSION PROMOTER COMPLEX
11	0.22	0.0	1.7	3.68	50	5	8	60	2o4a:A	60	84	☑	CRYSTAL STRUCTURE OF THE N-TERMINAL CUT DOMAIN OF SATB1 BOUND TO MATRIX ATTACHMENT REGION DNA
12	0.22	0.0	2.5	2.92	47	6	2	60	2e6w:A	60	96	☑	SOLUTION STRUCTURE AND CALCIUM BINDING PROPERTIES OF EF- HANDS 3 AND 4 OF CALSENILIN
13	0.22	0.0	1.1	4.11	49	4	20	60	2wiu:D	60	71	☑	MERCURY-MODIFIED BACTERIAL PERSISTENCE REGULATOR HIPBA
14	0.22	0.0	1.7	3.65	48	4	17	60	2vln:A	60	80	☑	N75A MUTANT OF E9 DNASE DOMAIN IN COMPLEX WITH IM9
15	0.22	0.0	1.9	3.58	48	4	17	60	2gzf:A	60	82	☑	CRYSTAL STRUCTURE OF THE E9 DNASE DOMAIN WITH A MUTANT IMMUNITY PROTEIN IM9 (Y54F)
16	0.22	0.0	1.9	3.59	48	4	17	60	2gze:A	60	82	☑	CRYSTAL STRUCTURE OF THE E9 DNASE DOMAIN WITH A MUTANT IMMUNITY PROTEIN IM9 (Y55A)
17	0.22	0.0	1.1	4.17	49	4	20	60	3dnv:B	75	71	☑	MDT PROTEIN
18	0.22	0.0	1.8	3.61	48	4	17	60	2vlp:A	60	82	☑	R54A MUTANT OF E9 DNASE DOMAIN IN COMPLEX WITH IM9
19	0.22	0.0	1.9	3.58	48	4	15	60	2gzj:E	60	83	☑	CRYSTAL STRUCTURE OF THE E9 DNASE DOMAIN WITH A MUTANT IMMUNITY PROTEIN IM9 (D51A)
20	0.22	0.0	1.9	3.58	48	4	15	60	2gyk:E	60	83	☑	CRYSTAL STRUCTURE OF THE COMPLEX OF THE COLICIN E9 DNASE DOMAIN WITH A MUTANT IMMUNITY PROTEIN, IMME9 (D51A)

b

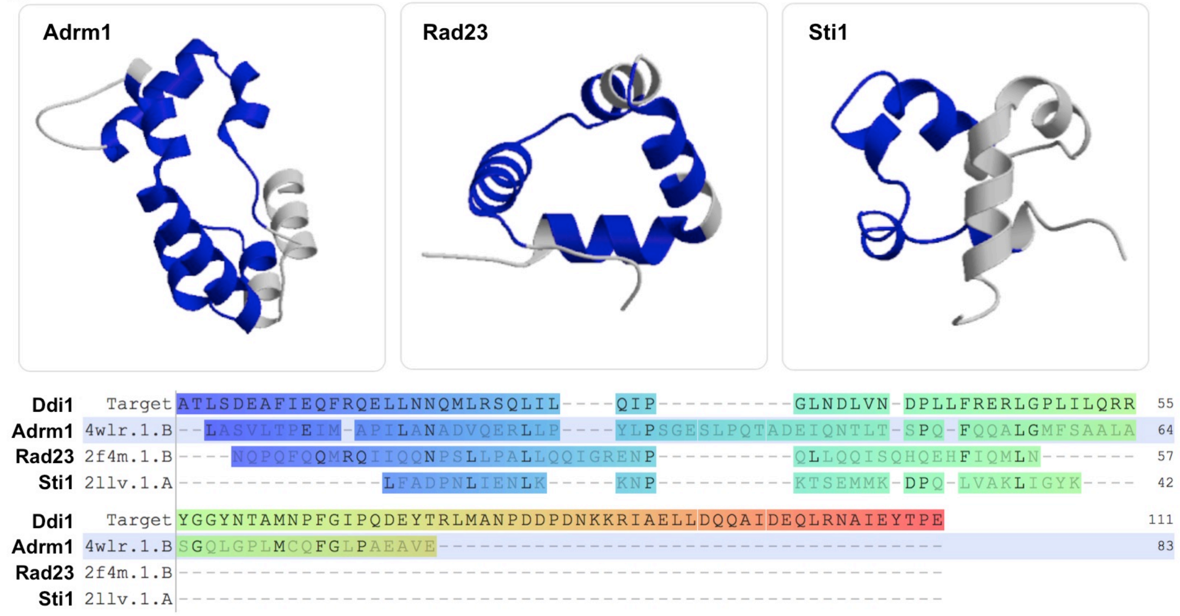

Supplementary Figure S7. Structural homology of the yeast HDD domain. **(a)** PDBeFold top hits for the HDD N-terminal domain (residue 90-141). Only structures with $N_{align} > 45$ were selected. **(b)** Output from the SWISS-MODEL web platform (http://swissmodel.expasy.org), using the yeast Ddi1 HDD sequence (86-196) as input. The three templates with the highest sequence identity (above 20%) were selected for analysis, excluding redundant structures with highly similar sequences. Top: structures of the three templates, with regions homologous to Ddi1 HDD highlighted in blue. Bottom: sequence alignment of Ddi1 HDD with the three templates, with PDB names indicated.

a

	Ddi1 UBL-HDD-RVP	Ddi1 HDD-RVP	Ddi1 HDD-RVP + 5% glycerol	Ddi1 RVP
R_g (SAXS)	40.7 ± 0.5 Å	40 ± 1 Å	34.6 ± 0.3 Å	25.9 ± 0.2 Å
R_h (QELS)	45 ± 3 Å	43 ± 4 Å	41 ± 4 Å	n.d.
M.wt (SAXS)	70 ± 5 kDa	65 ± 5 kDa	58 ± 5 kDa	35 ± 3 kDa
M.wt. (MALS)	72 ± 2 kDa	60 ± 4 kDa	52 ± 3 kDa	n.d.
Calc. M.wt	74.1 kDa	54.5 kDa	54.5 kDa	32.6 kDa

b

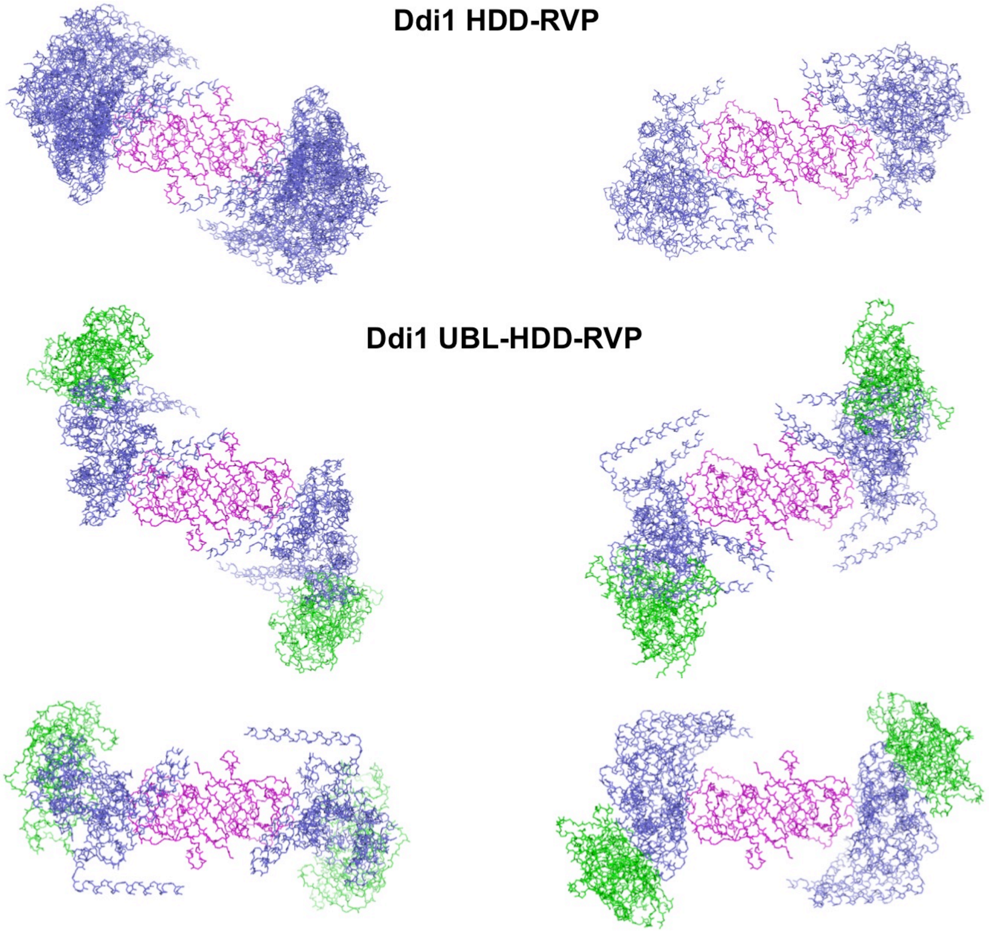

Supplementary Figure S8. SAXS analysis of Ddi1 HDD-RVP and UBL-HDD-RVP. (a) Summary of light (R_h, M.Wt) and small-angle X-ray (Rg, M.Wt) scattering parameters for different Ddi1 constructs under different conditions. N.d., not determined **(b)** Structure ensembles (twenty models) generated by the program CORAL for SAXS data acquired on Ddi1 86-325 (HDD-RVP) and 2-325 (UBL-HDD-RVP), using the UBL an HDD NMR structures, and the RVP dimer crystal structure. The UBL, HDD and RVP domains are colored in green, blue and magenta, respectively. The structures were sorted in two (HDD-RVP) or four (UBL-HDD-RVP) classes based on their similarities. The structures were superposed to the RVP domain, which was fixed during the calculations to maintain P2 symmetry. The average χ^2 for the HDD-RVP and UBL-HDD-RVP models are 1.36 and 1.19, respectively.

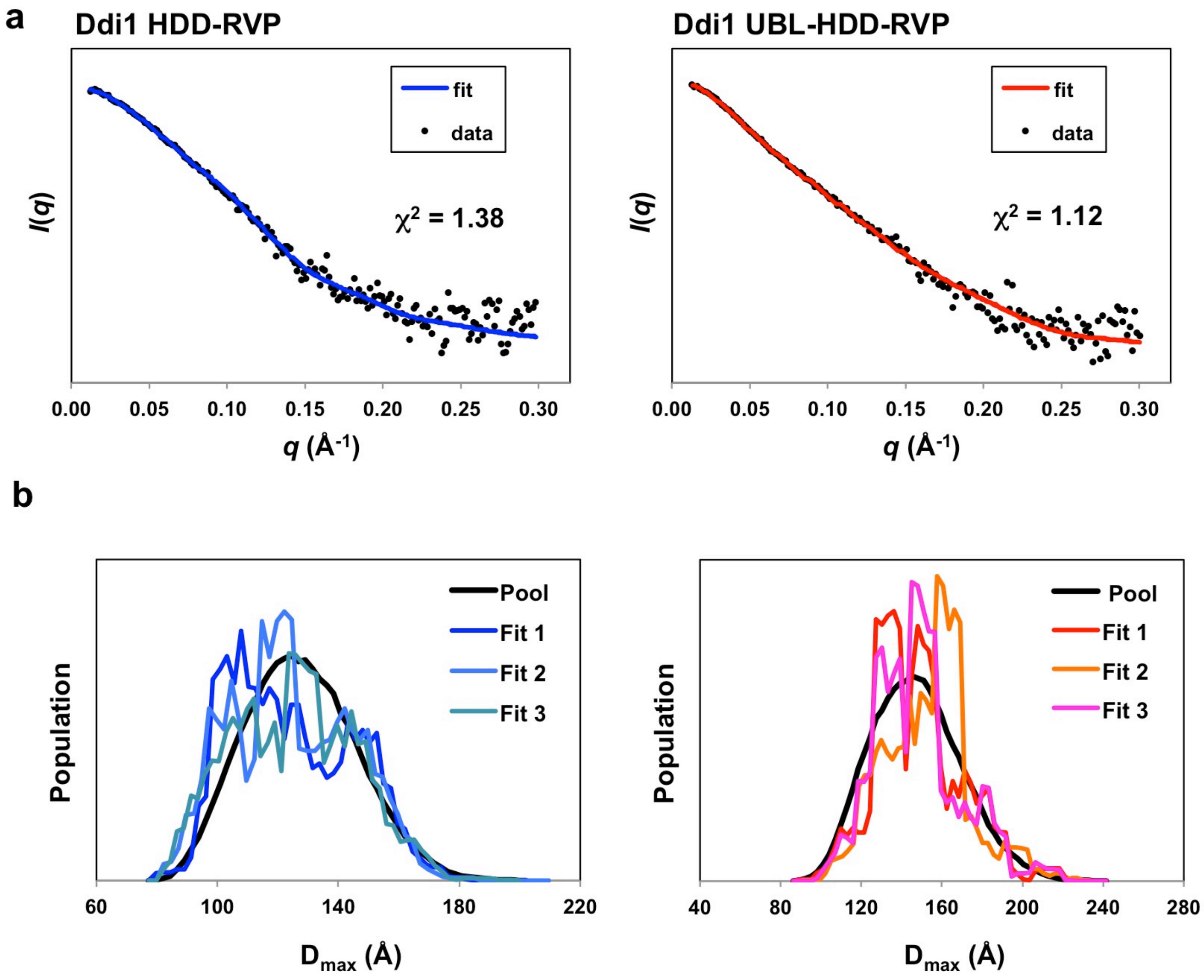

Supplementary Figure S9. Ensemble modeling of SAXS data for Ddi1 HDD-RVP and UBL-HDD-RVP. (a) Fit of the best ensemble of EOM-generated structures to experimental data for HDD-RVP (left) and UBL-HDD-RVP (right). **(b)** Population distribution of D_{max} for a pool of random structures of the UBL, HDD and RVP (black), compared with a ensembles that best fit the experimental data (blue and red for HDD-RVP and UBL-HDD-RVP, respectively).

Supplementary Table 1. X-ray diffraction data and refinement statistics for Ddi1 185-325

Data collection	CHESS A1
Wavelength (Å)	0.9789
Space group	$P2_12_12_1$
Unit cell dimensions	a=41.52 Å, b= 50.05 Å, c=131.56 Å
	α=β=γ=90°
Mosaicity (°)	0.30
Images	240
Oscillation angle (°)	0.5
Resolution (Å)	50.0-1.80 (1.90-1.80)
Unique reflections	25235 (2930)
Completeness (%)	96.1 (79.4)
Multiplicity	4.2 (2.6)
$\langle I/\sigma(I)\rangle$	25.3 (7.0)
R_{merge}†	0.036 (0.127)
Solvent content (%)	45
No. of reflections in R_{free} set	1285
R_{work}	0.183
R_{free}	0.213
FOM	0.881
Rms deviations from ideal values‡	
Bond length (Å)	0.011
Bond angle (°)	1.5
Torsion angle (°)	6.3
Protein atoms	2090
Solvent atoms	149
Average B-factor (Å^2)	
Protein main chain	23
Protein side-chain	26
Water	33
Ramachandran outliers§	none

† $\sum_{hkl}\sum_{i}\frac{|I_i(hkl) - \langle I(hkl)\rangle|}{\sum_{hkl}\sum_i I_i(hkl)}$, where $I_i(hkl)$ is the intensity of the *i*th measurement of reflection *hkl* and $\langle I(hkl)\rangle$ is the mean value for all *i* measurements

‡ Ideal values as reported in Engh & Huber (2001)

§ Residues for which the backbone torsion angles are outside the core region of the Ramachandran plot (Kleywegt & Jones, 1996).

Supplementary Table 2: NMR constraints and structural statistics for the Ddi1 UBL domain (residues 1-80)

**NMR distance & dihedral constraints**		
Distance constraints		
Total NOE	1653	
Intra-residue	490	
Inter-residue	1163	
Sequential (|i-j| = 1)	409	
Medium-range (|i-j| < 4)	290	
Long-range (|i-j > 5)	464	
Total dihedral angle restraints	128	
phi	64	
psi	64	
**Structure Statistics**		
Violations (mean and s.d.)		
Max. dihedral angle violation (º)	7.6	
Max. distance constraint violation (Å)	0.497	
Deviations from idealized geometry		
Bond lengths (Å)	0.012	
Bond angles (º)	1.4	
Ramachandran plot summary		
Most favoured regions	94.0%	
Additionally allowed regions	6.0%	
Generously allowed regions	0.0%	
Disallowed regions	0.0%	
Average pairwise r.m.s.d. (Å)	*ordered*	*all residues*
Heavy	0.7	2.0
Backbone	0.3	1.6

Supplementary Table 3: NMR constraints and structural statistics for the Ddi1 HDD domain (residues 86-196)

**NMR distance & dihedral constraints**		
Distance constraints		
Total NOE	1938	
Intra-residue	589	
Inter-residue		
Sequential (|i-j| = 1)	480	
Medium-range (|i-j| < 4)	514	
Long-range (|i-j > 5)	355	
Total dihedral angle restraints	168	
phi	84	
psi	84	
**Structure Statistics**		
Violations (mean and s.d.)		
Max. dihedral angle violation (º)	3.6	
Max. distance constraint violation (Å)	0.49	
Max. RDC constraint violation (Hz)	1.5	
Deviations from idealized geometry		
Bond lengths (Å)	0.002	
Bond angles (º)	0.452	
Ramachandran plot summary (90-141;151-187)		
Most favoured regions	96.4 %	
Additionally allowed regions	3.5 %	
Generously allowed regions	0.1 %	
Disallowed regions	0.0 %	
Average pairwise r.m.s.d. (Å)	*90-141*	*151-187*
Heavy	1.39	1.78
Backbone	0.57	0.99
Residual dipolar couplings restraints (N)	46	32
RMS deviation (Hz)	0.68±0.03	0.60±0.06
R-factor (%)	12.2±0.6	10.2±1.1
